# Metal–Organic Frameworks for Electrocatalytic Nitrate‐to‐Ammonia Conversion: A Review

**DOI:** 10.1002/open.70305

**Published:** 2026-10-01

**Authors:** Abdullah Shah, Muhammad Ilyas, Natasha Shehzad, Shah Faisal, Salman Khan, Nolubabalo Matinise

**Affiliations:** ^1^ Department of Chemistry Abdul Wali Khan University Mardan Mardan Pakistan; ^2^ UNESCO‐UNISA Africa Chair in Nanosciences/Nanotechnology College of Graduate Studies University of South Africa Pretoria South Africa

**Keywords:** ammonia electrosynthesis, charge‐transfer pathways, dynamic active sites, electrocatalytic nitrate reduction, metal–organic frameworks (MOFs)

## Abstract

This review takes a structure mechanism perspective on active site dynamics and charge transfer pathways governing ammonia selectivity in metal–organic frameworks (MOFs). This review aims to summarize the progress of MOF‐based electrocatalysts for nitrate to ammonia conversion, with a focus on structural characteristics, charge transfer behavior, and catalytic activity. The basic principles of nitrate reduction reaction (NO_3_^−^RR) such as reaction thermodynamics, reduction process, and important activity descriptors are presented. The recent developments on pristine MOFs, conductive MOFs, MOF‐derived materials and hybrid architectures are summarized and the roles of metal centers, ligand functionalization, defect engineering, heterometallic interactions and interfacial coupling in controlling nitrate adsorption, intermediate stabilization and hydrogenation processes in MOFs are highlighted. Representative MOF based systems achieve NH_3_ Faradaic efficiencies (FEs) up to about 99% with production rates near 22.5 mg h^−1^ cm^−2^ and nitrate conversion approaching 99%. The contribution of theoretical calculations and operando characterization techniques toward understanding reaction mechanisms is discussed. Key bottlenecks include low‐intrinsic conductivity, limited structural stability under reducing conditions, uncertain identification of the true active phase during reconstruction, and inconsistent testing protocols across studies. Finally, the current limitations and future research directions for developing stable, conductive, and practically relevant MOF‐based NO_3_^−^RR catalysts are critically evaluated.

## Graphical abstract

Electrocatalytic MOF‐based nitrate reduction transforms nitrate‐contaminated water into valuable ammonia. The process involves treating nitrate‐contaminated water. Engineering MOF catalysts with tunable structures. Activating dynamic metal sites within MOFs. Regulating charge transfer and proton availability. Facilitating multistep nitrate reduction pathways. Controlling competing reactions like hydrogen evolution. Achieving selective and efficient ammonia production.

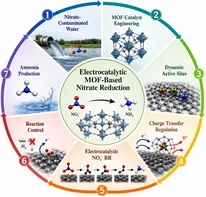

## 1 Introduction

Over recent decades, freshwater scarcity has intensified owing to accelerating water demand and pollution expansion, increasing the water stress from 14% to 58% in the early 20th century (approx. 200 million–3.8 billion) [1, 2]. Water contamination originates from various anthropogenic inputs, including industrial effluents, wastewater treatment facilities, landfill seepage, agricultural drainage, urban stormwater discharge, and atmospheric deposition, accompanied by the accumulation of heavy metals, persistent organic contaminants, pesticides, chlorination by‐products, and pharmaceutical residues [3, 4]. Rapid and poorly regulated urban development, demographic growth, and the release of untreated municipal, industrial, and agricultural wastes, together with landfill leachates, have led to widespread groundwater contamination [5]. Over the past 5 decades, nitrate (NO_3_^−^) contamination has emerged as a globally significant environmental issue and a major challenge for sustainable water resource management, representing one of the most prevalent forms of nonpoint source pollution associated with agricultural and rural activities [6, 7]. Increasing NO_3_^−^ levels in groundwater, mainly from agriculture, have been reported in various geographic regions around the world. The maximum contaminant concentration recommended by the United States Environmental Protection Agency (USEPA) for nitrate in drinking water is 45 mg L^−1^ [8]. The World health organization has reaffirmed the guideline of 50 mg L^−1^ for NO_3_^−^in drinking water, which is set to prevent the development of methemoglobinemia and other health impacts [9, 10, 11, 12, 13]. Excessive use of NO_3_^−^fertilizers and poor management of human and animal excrement are the main causes of nitrate contamination. Also, several industrial processes produce nitrate, such as the production of pulp and paper and munitions manufacturing [14]. The major source is agricultural practice, especially the extensive application of livestock manure and nitrogenous fertilizers, with sewage effluents as a minor source [15]. Because it is highly soluble in water and has a short soil residence time, NO_3_^−^ leaches easily into the subsoil when it is not taken up by plants. Besides, NO_3_^−^is highly mobile in aquifer systems and does not appreciably bind to mineral surfaces or precipitate [16]. Drinking water with a high NO_3_^−^ concentration could be a major environmental and public health problem for certain groups, especially infants, who are more vulnerable to the harmful effects of NO_3_^−^ toxicity [17, 18, 19]. Ingestion of nitrate‐contaminated potable water has emerged as an increasing public health problem as ingested nitrate can be metabolized to nitrite which can subsequently undergo nitrosation reactions to produce *N*‐nitroso compounds, a group of known carcinogens. There is an epidemiological association between nitrate exposure from drinking water and increased incidence of non‐Hodgkin's lymphoma (NHL), colorectal cancer, and methemoglobinemia [20]. These widespread sources, high environmental mobility, and associated health risks establish nitrate contamination as an important target for effective water remediation strategies. Currently, conventional biological nitrogen removal technologies, such as coupled nitrification–denitrification processes, can reduce nitrate to dinitrogen (N_2_) [21, 22, 23]. Although these approaches effectively remove nitrate from contaminated water, conversion to N_2_ results in the loss of nitrogen as a potentially valuable resource. Recently, selective reduction of nitrate to ammonia (NH_3_/NH_4_^+^) has been proposed as an alternative remediation strategy that has gained attention in the field of environmental engineering and resource recovery [24, 25, 26, 27, 28, 29, 30], attracting increasing attention in environmental engineering and resource recovery. Ammonia serves not only as a fundamental precursor for fertilizer manufacture but also as a prospective carbon‐neutral energy carrier [31]. Thus, the conversion of NO_3_^−^‐containing wastewater into ammonia provides an opportunity to simultaneously address nitrate pollution and utilize nitrogen as a valuable product. The attractiveness of ammonia recovery is further strengthened by the energy and environmental burden associated with conventional ammonia production. Industrial ammonia synthesis, however, is still dominated by Haber–Bosch process, which requires extremely harsh conditions (400–500 °C, 15–25 MPa) and is largely dependent on fossil feedstocks [32, 33, 34]. This process takes about 1–2% of global energy production every year and accounts for significant CO_2_ emissions [35, 36]. Consequently, the development of efficient ammonia generation technologies from NO_3_‐containing wastewater, driven by renewable electricity or solar energy, offers a promising approach to simultaneously alleviate environmental pollution and enhance nitrogen resource utilization, thereby advancing the realization of a sustainable circular nitrogen economy [37, 38, 39, 40]. In this context, electrocatalytic nitrate reduction (NO_3_^−^RR) provides a direct route for coupling nitrate remediation with ammonia production. In recent years, electrocatalytic nitrate reduction (NO_3_^−^RR) has become a promising green and environmentally friendly approach for ammonia synthesis under ambient conditions [41]. In this, the water is used as a proton and electron source, and the nitrate‐containing wastewater is adopted as a nitrogen feedstock, making green ammonia synthesis possible with high sustainability and low carbon emissions, efficient energy use, and minimal environmental footprint [42, 43]. NO_3_^−^RR will proceed through several coupled proton–electron transfer steps, and the overall pathway is highly sensitive to the adsorption energies of key intermediates on the surface [44]. The reaction starts with the adsorption of NO_3_^−^ on catalytically active sites. The first step, which is usually the rate‐determining step (RDS), is the nitrate deoxygenation reaction **(Reaction 1)** to produce nitrite (NO_2_^−^). The surface‐bound NO_2_ species is then reduced further and protonated to either gaseous nitric oxide (NO) or surface‐bound NO (*NO), which is required as an intermediate. The reaction pathway then splits into several mechanistic pathways at this point. The dominant pathway to NH_3_ is the stepwise hydrogenation of surface‐bound NO to hydroxylamine (NH_2_OH), followed by a subsequent proton–electron transfer, breaking the N─O bond, to produce NH_3_. However, this favorable sequence is involved in competition with parallel reactions. The overstrong binding of NO on the catalyst surface favors the N–N coupling pathway, resulting in N_2_ formation via N–N or NO–N intermediates, which reduces the ammonia selectivity [45, 46, 47, 48, 49]. By contrast, if the adsorption interaction is too weak, the activation of the intermediate is not complete, which decreases the catalytic performance [50, 51].



(1)

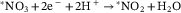




The overall performance of the reaction network is further controlled by the dynamic balance between controlled by the dynamic balance between generation and consumption of surface hydrogen species (H). The formation rate of these hydrogen species is typically linked to the dissociation of water or proton reduction, while the hydrogenation demand of each intermediate reaction, such as NO_3_^−^ must match the hydrogen coverage. Hydrogen coverage more than the hydrogenation demand will increase the hydrogen evolution reaction (HER), which competes with NO_3_^−^RR for active sites and electrons and significantly decreases FE. These competing pathways demonstrate that the central challenge in NO_3_^−^RR is not simply nitrate activation but the coordinated regulation of active sites, intermediate adsorption, proton–electron transfer, and surface hydrogen availability. The ideal catalyst is expected to achieve a balance in activation of NO_3_^−^, rational tuning of intermediate adsorption energies to suppress N–N coupling, and controlled production and consumption of H to facilitate efficient and selective NH_3_ synthesis. Meeting these requirements has motivated the development of both noble‐metal and earth‐abundant catalytic systems [52]. The noble metals such as platinum (Pt), palladium (Pd), and ruthenium (Ru) exhibit outstanding activity for NO_3_^−^RR because of their double‐shell electron structures and the ability to dissociate water efficiently. For instance, Xiong et al. have reported a RuNi alloy nanostructure with an NH_3_ production rate of 27.88 mg mg_cat_^−1^ h^−1^ and an NH_3_ FE of 98.02% under neutral conditions, which has been attributed to the synergistic interactions at the atomic level between Ru and Ni [53]. Similarly, Li et al. prepared CuPd/Cu_2_O nanoflower‐like heterostructure with controlled Pd content and designed interfaces, with an NH_3_ FE of 97.8% and nitrate conversion of 99.3% under the wide range of concentration of NH_3_ from 20 ppm to 1400 ppm [54]. Although noble metal catalysts are more efficient, their high price and significant hydrogen evolution capacity hinder their large‐scale applications [55]. These limitations highlight the need for alternative catalyst architectures in which active sites and their surrounding reaction environments can be deliberately regulated. On the other hand, the nonnoble catalysts, especially metal‐organic frameworks (MOFs), have become an increasingly attractive catalytic architecture over the past decade [56, 57, 58]. Due to their uniform distribution of active sites and their excellent structural adjustability, MOFs offer effective ways to tackle these shortcomings of traditional catalysts in NO_3_^−^RR [59, 60, 61]. These facilitate cooperative interaction between several metal nodes for better hydrogen species generation and utilization, and also their confined porous environment stabilizes the key intermediates, thus providing improved activity and selectivity for reaction pathways [62].

MOFs consist of metal nodes and organic linkers and possess a very large specific surface area (Figure 1), tunable pore structure, and tunable chemistry that can be used to control the pore environment and active site chemistry of the material. The periodic metal‐ligand networks give rise to quasimolecular catalytic behavior, making MOFs a perfect catalyst between homogeneous and heterogeneous catalysis [63, 64]. These attributes have spurred interest in MOFs as electrochemical processes. MOFs have been extensively investigated for electrochemical nitrate reduction (NO_3_^−^RR) to ammonia due to their tunable framework and functional sites that can increase NO_3_^−^ adsorption and proton transfer under ambient conditions [65, 66]. In practice, MOF‐derived electrocatalysts often achieve remarkably high NO_3_^−^RR efficiencies and selectivities, which is attributed to synergistic effects between the framework high surface area and dispersed active centers [67, 68, 69]. For instance, the high performance of conductive MOFs containing a lot of isolated metal atoms (e.g., Cu or Fe) for NO_3_^−^‐to‐ammonia conversion under neutral conditions has been reported [67]. The ability of MOFs to regulate the local reaction environment has been demonstrated experimentally.

**FIGURE 1:**
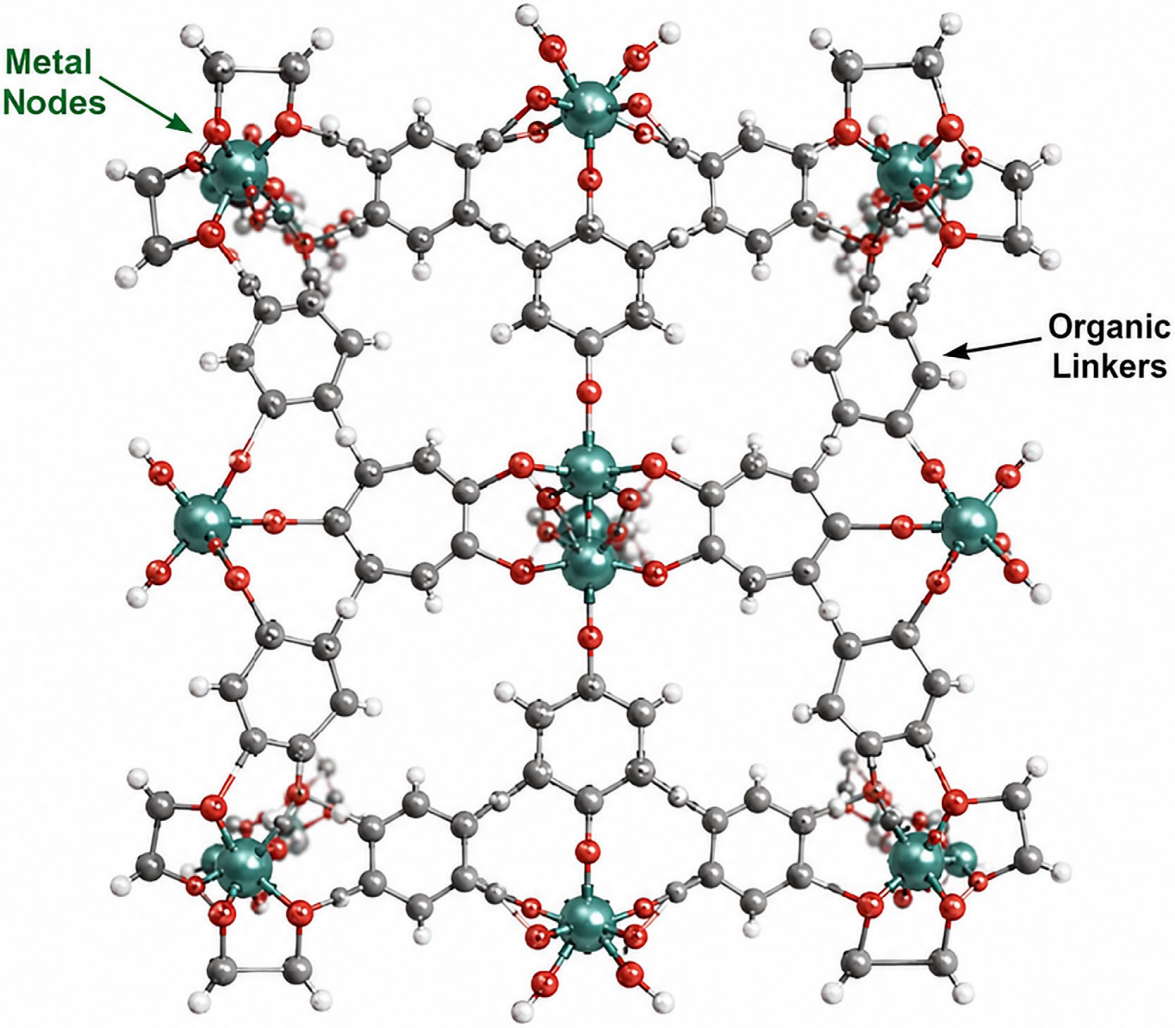
Schematic of MOF showing metal nodes and organic linkers.

For instance, a proton‐rich interlayer was constructed by coating the Cu electrode with a sulfonate‐functionalized Zr‐MOF (SO_3_‐MOF‐808), and the NH_3_ FE increased from 75% (bare Cu) to 87.5%. This shows the ability of a highly porous, anionic MOF layer to focus protons at the catalyst interface and inhibit competing nitrite formation in NO_3_^−^RR [70]. Key features making MOFs unique electrocatalysts include their exceptionally large electrochemically active surface area (ECSA) and the ability to host isolated metal sites within well‐defined pores [67, 71]. Moreover, the local electronic environment can be tuned and key intermediates stabilized by integrated ligand functionalities (e.g., sulfonate groups or N‐containing moieties on linkers), thus allowing an effective control of adsorption energies [72]. These properties enable MOFs to exert quasi‐molecular control over catalytic pathways and thus tailor catalytic pathways for reaction kinetics and high catalytic turnover within the framework. Therefore, the high NO_3_^−^RR activity of many MOF catalysts can directly be attributed to their structure: Interconnected pore network can be able to enable quick transport of NO_3_ and H^+^ molecules, whereas built‐in functional groups (such as SO_3_^−^ or N^−^ centers) can ensure a focused concentration of reactants at the active site, which can further reduce the activation barrier for the generation of ammonia [66, 71]. Despite their benefits, there are certain limitations associated with MOFs, such as their relatively low intrinsic conductivities and their potential susceptibility to water‐based electrolytes [66, 73]. Even the most competent Zr‐based frameworks are rather isolated, allowing a slow charge to the active sites [66, 72]. Several strategies have been investigated to overcome these shortcomings, such as MOF design to make MOFs intrinsically conductive or the preparation of MOF/carbon composites [5]. Other approaches include employing stable MOFs (e.g. Zr─O bonds in UiO‐type MOFs) to enhance water stability and transform MOFs into conductive porous carbon or single‐atom catalysts via controlled pyrolysis [66, 71]. These activities allow for reduced weakening of the MOF, retaining the benefits, and produce highly efficient, long‐lived NO_3_^−^ to NH_3_ catalysts [66, 71]. MOFs permanent porosity and tunable chemistry have resulted in some of the most selective NO_3_^−^RR electrocatalysts known to date [65, 66]. Recently, such MOF‐based systems have achieved NH_3_ faradaic efficiencies of around 96% at high nitrate conversion, with NH_3_ production rates of 102 μmol cm^−2^ h^−1^ [69], showing competitive performance for sustainable ammonia synthesis [66].

However, the intrinsic activity and electrical conductivity of pristine MOFs are not high enough, and the structural strength of pristine MOFs is not robust enough for practical electrocatalytic systems. To overcome these limitations, two key modification strategies have been proposed: (1) tuning the electronic structure and distribution of MOFs by adding heterometals or preparing hybrid composites and (2) using MOFs as sacrificial templates for producing MOFs with hierarchical porosity and active centers with controlled pyrolysis [74, 75, 76, 77, 78, 79]. The combination of NO_3_^−^‐remediation requirements, ammonia resource recovery, the mechanistic complexity of NO_3_^−^RR, and the need for controllable catalytic environments therefore provides the basis for examining MOF‐based electrocatalysts from a structure‐mechanism perspective.

This review is a systematic summary of the recent advances in nitrate reduction (NO_3_^−^RR) to ammonia synthesis by MOFs‐based electrocatalysis, focusing on structure–activity relationships and charge‐transfer regulation mechanisms. This review emphasizes four key concepts for controlling catalytic activity and selectivity: microstructure engineering, electronic structure modulation, defect construction, and optimization of interfaces, as opposed to previously reported reviews which were mainly concerned with catalyst performance comparisons. Pristine MOFs, conductive MOFs, MOF‐derived nanostructures, and MOF‐based hybrid systems are discussed in detail, and the relationships between their structures and mechanistic pathways, intermediate stabilization, and hydrogen evolution suppression are discussed. Moreover, advanced in situ characterization techniques and calculations are combined to help elucidate the reaction kinetics and proton‐coupled electron transfer (PCET) processes. Lastly, critical challenges and future perspectives are suggested for the rational design of highly efficient, stable, and scalable MOF‐based catalysts for sustainable nitrate‐to‐ammonia conversion.

## 2 Fundamentals of Electrocatalytic Nitrate Reduction

For effective electrocatalyst development towards NO_3_^−^RR, it is essential to have a fundamental understanding of the fundamentals of the reaction. Now, the mechanism of this process is being explored using theory simulations and experiments. The following summarizes the major ingredients of electrolytic NO_3_^−^RR with an emphasis on some of the important key thermodynamic and kinetic factors.

### 2.1 Thermodynamics of NO_3_ to NH_3_ Conversion

NO_3_^−^reduction mediated by electrons in aqueous solution involves the transfer of electron and proton (proton coupled electron transfer), which usually give rise to N_2_ intermediates such as N_2_O, NO, NH_2_OH, and NO_2_ [80]. The oxidation states of nitrogen can vary from −3 to +5, leading to a complex network of thermodynamic hydrido and oxo species, as illustrated in the Forst–Ebsworth diagram (Figure 2a). This reaction sequence reduces NO_3_^−^ to NO_2_^−^ and then to various products including HNO_2_, NO, NH_2_O, NH_3_, N_2_O, and N_2_ [85]. The standard state thermodynamically most stable nitrogen species are N_2_ and NH_3_ by the Forst–Ebsworth diagram [86]. The Pourbaix diagram (Figure 2b) shows that such reduction processes occur close to the water decomposition limit, as a function of pH, applied potential, and NO_3_^−^ concentration [87, 88, 89]. The Frost–Ebsworth diagram plots the free energy of formation of nitrogen species (normalized by the number of nitrogen atoms) against the oxidation state at fixed pH. At both pH 0 and pH 14, the most stable end‐products are N_2_ and NH_3_/NH_4_^+^, while intermediate species (NO_2_^−^, NO, N_2_O, and NH_2_OH) lie higher in free energy and are therefore metastable [65]. The slope of each line segment is proportional to the standard reduction potential of the corresponding half reaction. Consequently, the diagram immediately reveals that the eight‐electron reduction of NO_3_^−^ to NH_3_ is thermodynamically less downhill than the 10‐electron reduction to N_2_ [80, 90].

**FIGURE 2:**
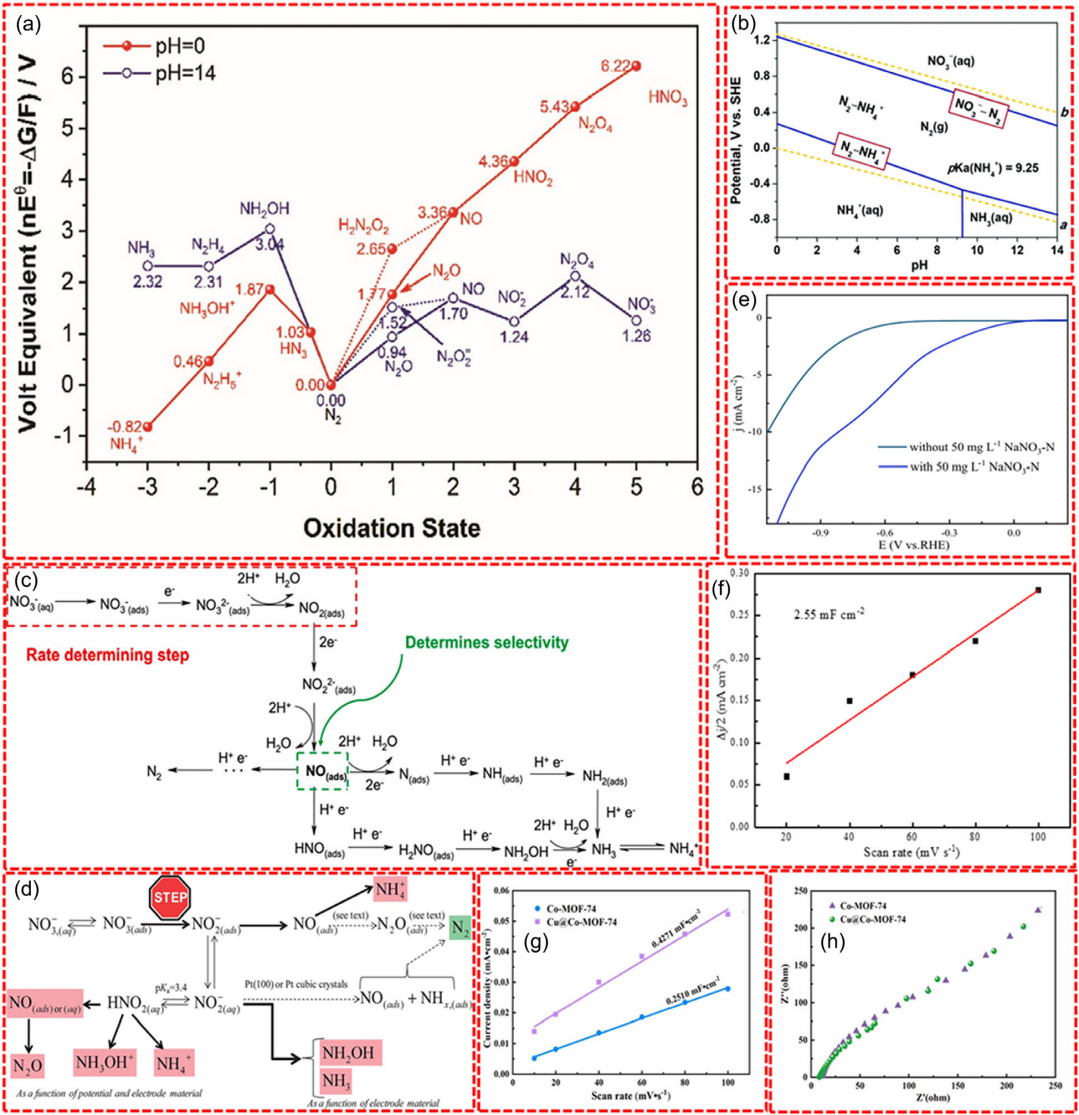
(a) Frost–Ebsworth diagram illustrating nitrogen species at pH 0.0 and pH 14. Reproduced with permission from [81]. Copyright 2020, Wiley. (b) Pourbaix diagram depicting nitrogen species. The solid lines represent redox reactions among NO_3_, N_2_, NH_3_, and NH_4_^+^. Dotted lines in (a) (HOR/HER) and (b) (OER/ORR) divide the water stability region. Reproduced with permission from [82]. Copyright 2022, Royal Society of Chemistry. (c) Direct surface‐mediated pathway under typical conditions, highlighting the RDS (*NO_3_ → *NO_2_; Reactions 6 and 7) and the PDS (*NO → *NOH/*HNO). Figure reproduced from [65], which is open access and permits unrestricted use of materials under the term of the Creative Common license CC‐BY, which is open access and permits unrestricted use of materials under the term of the Creative Common license CC‐BY. (d) Indirect autocatalytic route that becomes dominant under strongly acidic conditions and high nitrate concentrations. Reproduced with permission from [80]. Copyright 2012, Royal Society of Chemistry. (e) LSV curves for Co_0.1_@MIL‐Fe cathode in absence and presence of 50 mg/L, (f) linear fitting of capacitive currents of the catalysts versus scan rates. The double‐layer capacitance (C_dl_) correlates with the electrochemical surface area (ECSA). Reproduced with permission from [83]. Copyright 2021, Elsevier. (g) Co‐MOF‐74 and Cu@Co‐MOF‐74 current density difference and scan rate correspondence; (h) EIS characterization in a mixture of Co‐MOF‐74 and Cu@Co‐MOF‐74 with 0.1 M PBS and 50 mM KNO. Reproduced with permission from [84]. Copyright 2025, Elsevier.

The Pourbaix diagram translates these free‐energy relations into the E–pH plane. The equilibrium lines for NO_3_^−^/N_2_ (E° = +1.17 V vs. SHE) and NO_3_/NH_4_^+^ (E° = −0.12 V vs. SHE at pH 0) define the thermodynamic windows in which each product is favored. Thermodynamically, N_2_ possesses the highest equilibrium potential among possible NO_3_^−^/product couples and is therefore the preferred product under standard conditions [80, 91]. Because both pathways compete with the HER and the oxygen evolution reaction (OER), the practical operating window for selective NH_3_ production is narrow and strongly pH‐dependent [89, 90]. The standard potential for NH_3_ generation is −0.12 V versus SHE **(Reaction 3),** while the standard potential for N_2_ formation is 1.17 V versus SHE **(Reaction 4)** [65]. The total energy efficiency depends on the competition between cathodic reduction of NO_3_^−^ and anodic evolution of oxygen **(Reaction 5)** and hydrogen evolution [90]. Quantitative comparison of the standard potentials shows the thermodynamic cell voltage for the overall reaction:

(2) $${\mathrm{NO}}_{3}^{-}+H_{2}O\;\to \;{\mathrm{NH}}_{3}+\frac{1}{2}O_{2}+\frac{1}{2}H_{2}O\;\text{is only 1.35 V}(-0.12+1.23V)$$

In contrast, the competing nitrate‐to‐N_2_ pathway coupled to the OER requires a cell voltage of only 0.06 V. This large difference quantifies why N_2_ is the thermodynamically preferred product and why substantial kinetic control is required to obtain high FE for NH_3_ [80, 90, 91]. To achieve the practical benefits, NO_3_^−^RR‐based approaches combine NO_3_^−^RR with anodic oxidation of organic compounds, replacing the slow OER to generate value‐added chemicals and reduce HER**.** Computational analysis using DFT shows that the Gibbs free energy for NO_3_^−^ adsorption and deoxygenation on modified surfaces is more negative than that on the unmodified surfaces, with PDSs like the transition from *NO to *NOH having lower barriers (0.54 eV vs. 1.56 eV) due to the cooperation between the heteroatoms [62]. Similarly, in Fe–Co(OH)F/CO‐TEPB, the adsorption energy of NO_3_^−^ at the Fe sites (−2.20 eV) is higher than the adsorption energy at the Co sites (−1.66 eV), which is not favorable for the PDS, yet shows that the absorption energy is related to the electronic structure and thus plays an important role in determining thermodynamic favorability [92]. Under acidic conditions, the Pourbaix lines for many first‐row transition‐metal redox couples (including Cu^2+^/Cu^+^ and Fe^3+^/Fe^2+^) lie within or near the potential window of nitrate reduction, allowing partial reduction of the metal nodes during electrolysis. This valence change alters both the nitrate adsorption energy and the subsequent hydrogenation barriers [65, 89]. At near‐neutral to mild alkaline pH (typical of wastewater), many of these nodes remain in higher oxidation states, favoring the initial two‐electron reduction to nitrite, while the subsequent multi‐electron steps become kinetically more demanding. The net result is a pH‐tunable product branching: Higher proton activity under acidic conditions accelerates the eight‐electron pathway to NH_3_, whereas proton scarcity under alkaline conditions shifts selectivity toward N_2_ or residual NO_2_^–^ unless proton‐relay functionalities inside the MOF pores are deliberately engineered [89, 92, 93].

(3) $${\mathrm{NO}}_{3}+9H^{+}+8e^{-}\;\to \;{\mathrm{NH}}_{3}+3H_{2}O,\;E\hat{A}{}^{\circ}=-0.12\;\text{V vs SHE}$$

(4) $${\mathrm{NO}}_{3}+12H^{+}+10e^{-}\;\to \;N_{2}+6H_{2}O,\;E\hat{A}{}^{\circ}=1.17\;\text{V vs SHE}$$

(5) $$4{\mathrm{OH}}^{-}\;\to \;O_{2(g)}+2H_{2}O+4e^{-},\;E\hat{A}{}^{\circ}=1.2\;\text{V vs SHE}$$

(6) $$2H^{+}+2e^{-}\;\to \;H_{2(g)}\;E\hat{A}{}^{\circ}=0\;\text{V vs SHE}$$

[94]

Because the thermodynamic driving force for N_2_ is substantially larger than that for NH_3_, selectivity control inside MOF systems relies on kinetic rather than purely thermodynamic factors. The key branch point is the *NO intermediate [80, 95]. When the local proton supply is high and the metal node can stabilize *HNO or *NOH, the pathway proceeds to NH_3_. When *NO coverage is high and proton supply is limited, *NO can recombine with partially hydrogenated fragments (such as *NH_2_) in a Langmuir–Hinshelwood step to give N_2_ or dimerize to form N_2_O that is further reduced to N_2_ [95, 96]. MOF architectures that create a proton‐rich microenvironment or dual active sites (one for NO_3_^–^ activation and one for active hydrogen generation) therefore shift the branching ratio toward ammonia even though the thermodynamic preference remains for N_2_ [91, 92, 93].

### 2.2 Overall Reaction Stoichiometry and Electron Requirements

The conversion of NO_3_^−^ to NH_3_ proceeds through an eight‐electron, nine‐proton‐transfer process **(Reaction 3)** [86]. In contrast, the formation of N_2_ demands ten electrons and 12 protons **(Reaction 4)** [86]. The multielectron nature of the system is evident in these reactions, which show that the products of the reactions are dependent on the pH; below a pH of 9.25, NH_4_^+^ is produced, and above a pH of 9.25, NH_3_ (aq) is produced [97]. The process is heterogeneous with many oxidation state changes between +5 and −3, resulting in several different products that are strongly dependent on reaction conditions [98, 99, 100]. The reaction initiates with NO_3_^−^ adsorption (*NO_3_), followed by the RDS **(Reaction 7)** [87].

This reaction is often rate limited by the formation of *NO_2_ **(Reaction 8)** with Tafel slopes near 120 mV per decade on metals including Ru, Rh, Ir, Pt, Cu, and Ag and then branches into two reactions, one to form *NO and the other to either NH_3_ or N_2_ [95, 101, 102, 103]. In a very acidic solution with a high level of NO_2_^−^, indirect pathways (via HNO_2_ or NO_2_) dominate, while direct pathways dominate in more typical environments [89, 104, 105]. The necessity of catalysts with the right adsorption properties for NO_3_^−^ while keeping the surface free from poisoning by other species, such as Cu sites of high adsorption capacity for NO_3_^−^ but that may be less efficient for hydrogenation unless supported by proton‐assisting sites like Co, is emphasized in the electron demand [84].



(7)

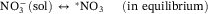






(8)

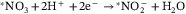




### 2.3 Stepwise Reduction Pathways

Electrocatalytic NO_3_^−^RR proceeds through two principal routes whose relative importance depends on electrolyte conditions, and both routes converge on the critical *NO intermediate that controls the selectivity between NH_3_ and N_2_^–^. Two dominant mechanisms operate.

#### 2.3.1 Direct Pathway

Under typical conditions ([NO_3_^–^] < 1 M, moderate acidity or near‐neutral pH), reaction 7 a surface‐mediated sequence (Figure 2c).The reduction of adsorbed nitrate to nitrite (*NO_3_ → *NO_2_^–^, reactions 8) is the RDS in most systems [80, 87, 104]. *NO_2_ is further converted to *NO, after which the hydrogenation of *NO to *NOH or *HNO occurs. This hydrogenation step is identified as the PDS in most DFT profiles. Subsequent hydrogenation proceeds through *NH_2_OH to *NH_3_ **(Reaction 9)** [65, 104, 106]. A commonly proposed sequence is *NO_3_ → *NO_2_ → *NO → *NOH → *NH_2_O → *NH_2_OH → *NH_2_ → *NH_3_ [92]. Spectroscopic evidence supporting these intermediates includes FTIR signals of NO_2_ (1260 cm^−1^), NO (1700 cm^−1^), and NH_2_OH (1155 cm^−1^) [92, 107, 108].

#### 2.3.2 Indirect Autocatalytic Route

At high acidity and high nitrate concentration (>1 M NO_3_^−^, potentials ≈ 0.8−0.9 V vs. SHE), a homogeneous autocatalytic cycle involving HNO_2_/NO_2_ becomes dominant [65, 89, 105]. In this route, nitrite generated in solution is protonated to HNO_2_ and further reduced, accelerating the overall conversion while still feeding surface *NO intermediates. Once *NO is formed, two competing channels open (Figure 2d) [65, 80, 86]. The NH_3_ channel proceeds through sequential hydrogenation (*NO → *NHO/*NOH → *H_2_NO/*NH_2_O → *NH_2_OH → *NH_2_ → *NH_3_), corresponding to the overall eight‐electron pathway **(Reaction 3)** and is favored by high local proton activity and stabilization of *HNO/*NOH. The N_2_ channel instead proceeds through *NO dimerization to N_2_O followed by further reduction or through Langmuir–Hinshelwood coupling of *NO with partially hydrogenated fragments (*NH_*x*_), corresponding to the overall 10‐electron pathway (Reaction 4), and is favored by low proton supply and high *NO coverage [95, 96]. Hydrogen‐assisted routes involve *H‐mediated processes (Volmer step) and PCET; PCET can bypass *H adsorption, thereby suppressing the competing HER and enhancing NH_3_ selectivity at high current densities [93, 109].

#### 2.3.3 Differentiation of RDS and PDS

Experimental Tafel slopes near 120 mV dec^–1^ on a wide range of metals and MOF‐derived catalysts identify the first two‐electron deoxygenation (*NO_3_ → *NO_2_^–^), **(Reactions 8)** as the RDS that limits the overall kinetic rate [95, 101, 102, 103]. In contrast, density functional theory free‐energy profiles show that the subsequent hydrogenation of *NO to *NOH (or *HNO) carries the largest thermodynamic barrier after the initial deoxygenation; this step is therefore the PDS that sets the theoretical overpotential [80, 110, 111, 112]. The RDS controls the observed current density at a fixed potential, while the PDS determines the minimum overpotential required for high FE toward NH_3_ [65, 86].

#### 2.3.4 Correlation of MOF Structural Features With RDS and PDS Energy Barriers

The MOF architecture provides multiple structural handles to lower both the RDS and the PDS barriers [62, 92, 112]. Because MOFs are constructed by self‐assembling discrete metal or metal‐cluster nodes with organic linkers into a periodic, crystallographically well‐defined lattice, the identity of the active site, the chemical environment surrounding it, and the arrangement of neighboring active sites can in principle be varied independently, a degree of modular control that is difficult to achieve in conventional heterogeneous catalysts, where composition, coordination environment, and surface structure are typically coupled [72, 113]. This modularity allows the RDS and PDS to be targeted by distinct, largely orthogonal structural strategies rather than by a single composition–activity relationship, and these such strategies are examined in turn below. First, the identity of the metal node encompassing its elemental composition, oxidation state, and nuclearity sets the intrinsic d‐band position and adsorption energetics of *NO_3_^−^, *NO_2_^−^, and *NO, and second, functionalization of the organic linker and the resulting secondary coordination sphere modifies the local electrostatic and hydrogen‐bonding environment around the active site without altering the metal node itself, and third, incorporation of a second metal or an engineered phase boundary redistributes electron density across neighboring active sites through d‐band engineering. A further, complementary handle operates at the level of the pore rather than the active site: well‐defined MOF channels can act as smart, size‐ and charge‐selective conduits that concentrate NO_3_^−^ near the active site and regulate the local residence time of reaction intermediates, thereby influencing the RDS and PDS indirectly through mass transport rather than purely electronic effects [114]. Hierarchically porous MOF architectures extend this principle by combining accessible macro‐/mesoporosity for facile NO_3_^−^transport with a high density of microporous active sites, illustrating that pore architecture and active‐site electronic structure are complementary, rather than competing, design considerations [115].

#### 2.3.5 Metal‐Node Identity and Electronic Structure

Fe sites typically exhibit stronger *NO_3_ adsorption than Co sites, facilitating the initial deoxygenation RDS **(Reaction 8)**. Co sites, in contrast, supply active hydrogen more readily and lower the barrier of the *NO → *NOH PDS. In Fe–Co dual‐site systems, the two metals act synergistically, reducing the PDS barrier from 1.56 eV on monometallic references to 0.54 eV [62, 92, 116]. Beyond the Fe−Co pair, the electronic structure of metal nodes varies systematically across the first‐row transition‐metal series and correlates with *NO_3_ adsorption and NO_3_^‐^RR activity. Fe and Co generally bind *NO_3_^−^ and *NO more strongly than later 3d metals such as Ni and Cu, reflecting differences in d‐electron count and oxidation state [104]. Local doping can further tune this behavior; for example, B incorporation into Cu‐based MOF nodes withdraws electron density from Cu 3d orbitals, downshifts the d‐band, weakens competing *H adsorption, and thereby improves NH_3_ selectivity [107]. DFT screening further identifies the d‐band center, rather than the nominal oxidation state alone, as a key descriptor of *NO_3_, *NO_2_^−^, and *NO adsorption, providing a unified explanation for the complementary Fe‐ and Co‐type roles in RDS/PDS energetics [110]. Metal‐node nuclearity and connectivity provide an additional electronic structure parameter. Square‐planar tetranuclear coordination polymers redistribute charge among closely coupled metal centers, stabilizing multiple nitrogen intermediates, and promoting nitrate‐to‐ammonia conversion [114]. Similarly, increasing nuclearity in cobalt‐cluster coordination polymers lowers the hydrogenation PDS barrier through cooperative hydrogen supply from adjacent Co centers [117]. Heterometallic Fe_2_M clusters further tune redox properties through central‐metal substitution while retaining the Fe_2_M topology, sustaining NO_3_^−^RR activity under acidic conditions [118]. Thus, cluster nuclearity and topology provide electronic structure control beyond elemental substitution. Coupling MOF nodes with noble metals introduces another route for tuning the hydrogenation chemistry. Ir nanoclusters embedded in Co‐MOF nanosheets promote charge transfer from Co to Ir, enhancing *NO_3_^−^ activation at Co while facilitating *H generation and *NOH/*HNO hydrogenation at Ir, thereby lowering the RDS and PDS barriers [119]. Ru_*x*_O_*y*_ clusters similarly divide catalytic functions between nitrate activation at the MOF node and proton‐coupled electron transfer at Ru sites [120]. Noble‐metal nanodots on redox‐active MOF nodes further demonstrate that interfacial charge transfer, controlled by nanodot size and node redox state, regulates *NOH adsorption and therefore acts as a tunable electronic structure descriptor [121]. Importantly, the metal‐node electronic structure evolves under operating conditions. In Cu‐based MOF/CuPc systems, Cu^2+^ nodes are progressively reduced to Cu^+^/Cu^o^C species, with the resulting lower valent phase serving as the active state responsible for high NH_3_ selectivity [122]. Likewise, isolated Cu single atoms confined in MOF pores can cluster into subnanometric Cu ensembles during electrolysis, generating intermediate coordination environments that favor *NO hydrogenation over N–N coupling [123]. These observations demonstrate that the as‐synthesized node may not represent the true catalytic state and highlight the importance of monitoring the oxidation state and coordination environment during operation. Overall, metal dilution with a second 3d metal, cluster nuclearity, noble‐metal decoration, and operando valence reconstruction provide complementary strategies for tuning the metal‐node electronic structure. Other Fe‐ and Co‐based systems support this concept: Co‐doped Fe/Fe_2_O_3_ nodes combine strong Fe^3+^–*NO^3−^ interactions with Co‐induced hydrogenation activity [124]; Fe(III) incorporation into MOF‐808 with phosphate co‐adsorption modifies the Fe environment and promotes proton shuttling [125]; while low‐crystallinity Co−N−C phases derived from Co‐MOF reconstruction generate under‐coordinated Co sites that favor *NOH stabilization over N_2_ evolution [126, 127]. Collectively, these studies establish metal‐node identity including composition, oxidation state, nuclearity, and dynamic valence as a key electronic structure descriptor governing RDS and PDS energetics.

#### 2.3.6 Ligand Functionalization and Secondary Coordination Sphere

Methyl‐functionalized ligands (DiMe‐Cu_3_‐MOF) form hydrogen bonds with *NO_2_, lowering the free‐energy change for *NO_2_ → *NO_2_H from –0.66 eV to –0.98 eV. This thermodynamic assistance propagates through subsequent steps and reduces the effective barrier of the PDS [112]. Proton relay groups inside the MOF pores further increase local proton activity, preferentially accelerating the hydrogenation PDS over competing hydrogen evolution [93, 128]. Beyond ligands that engage individual adsorbed intermediates through direct hydrogen bonding, functional groups in the secondary coordination sphere can be introduced through pre‐ or postsynthetic modification of the organic linker, providing a general and modular route to tune the pore microenvironment independently of the metal node. Pore‐engineering strategies that decorate MOF channels with coordinating or hydrogen‐bond‐donating functionalities (e.g., ─NH_2_, ─OH, ─SO_3_H) alter the local electrostatic field and polarity experienced by adsorbed nitrogen oxyanions without changing the coordination geometry of the metal node itself [129]. Because many MOFs retain their crystalline topology upon linker exchange, postsynthetic ligand exchange and postsynthetic functionalization allow a single parent framework to be systematically decorated with a panel of functional groups, enabling structure–activity relationships for the secondary coordination sphere to be established largely independent of node‐level effects [130, 131]. This modularity is illustrated by sulfonate‐functionalized MOFs, in which pendant –SO_3_H groups lining the pore walls behave as a porous proton reservoir: The acidic sulfonate protons dissociate readily under operating conditions and are shuttled to nearby adsorbed nitrogen intermediates, sustaining locally elevated proton activity even as the bulk electrolyte is depleted near the electrode surface. This buffering capacity is proposed to underlie the enhanced NO_3_^−^RR to NH_3_ activity reported for sulfonate‐decorated frameworks relative to their unfunctionalized parent MOF [70], giving the proton‐relay mechanism described above [93, 128] a concrete structural motif that can be installed by rational linker design. The secondary coordination sphere also governs how the framework itself responds mechanically and electronically to adsorbate binding. In flexible MOFs, adsorption‐induced lattice distortion around the metal node a pseudo‐Jahn Teller‐type effect locally perturbs the coordination geometry and electronic structure of the active site as reaction intermediates bind and desorb, and this dynamic structural flexibility has been shown to lower the overall barrier for electrochemical nitrate reduction relative to rigid, nondistorting analogues [132]. Such linker flexibility effects indicate that the secondary coordination sphere is not simply a static scaffold decorated with fixed functional groups but can itself actively participate in accommodating the geometric and electronic demands of the reaction intermediates along the RDS to PDS pathway, complementing the operando node reconstruction. The deliberate introduction of linker vacancies provides a further route to reshape the secondary coordination sphere. Defect engineering of Cu‐based MOFs generates missing‐linker sites that expose additional open coordination positions on the metal node and locally reorganize the surrounding hydrogen‐bonding network, together increasing the density of accessible active sites and improving nitrate‐to‐ammonia selectivity relative to the defect‐free framework [133]. Because defect density and distribution can be controlled synthetically, for example, through modulator concentration or activation conditions, defect engineering offers a tunable complement to covalent ligand functionalization for optimizing the local pore environment around the active site. Ligand identity additionally governs charge transfer in more complex tandem MOF architectures. In MOF/copper‐phthalocyanine tandem electrocatalysts, varying the organic ligand alongside the Co/Cu metal ratio was found to modulate the electronic coupling between the MOF and the phthalocyanine cocatalyst, indicating that ligand choice and metal‐node composition act as coupled, rather than independent, design variables in multicomponent systems [134]. Collectively, functional group installation, proton‐reservoir chemistry, linker flexibility, and defect engineering represent complementary strategies for tuning the secondary coordination sphere; together with the metal‐node electronic structure descriptors, they define the two principal molecular‐design axes through which MOF architectures lower the RDS and PDS barriers for NO_3_^−^RR to NH_3_.

#### 2.3.7 Bimetallic Interfaces and d‐Band Engineering

Incorporation of a second metal (Cu–Co, Cu–Ni, and Fe–Co) shifts the d‐band center closer to the Fermi level (e.g., from –3.47 eV to –3.22 eV) in Cu_1_Co_1_HHTP. The resulting stronger adsorption of nitrogen intermediates simultaneously facilitates N–O bond cleavage (RDS) and stabilizes *NOH/*HNO (PDS) [62]. In optimized Cu–Ni alloys, the PDS switches from NO_2_ hydrogenation (0.39 eV on pure Cu) to *NO hydrogenation (0.27 eV), suppressing NO_2_^–^ release and raising NH_3_ selectivity [135]. The d‐band shifts identified for Cu−Co and Cu−Ni MOF nodes reflect a more general principle of alloy electronic structure that extends beyond MOF chemistry. Atomic‐scale cooperativity between neighboring metal atoms in bimetallic alloy nanostructures has been shown to redistribute charge density in a manner that simultaneously optimizes multiple adsorption steps of the nitrate‐to‐ammonia pathway rather than merely shifting a single descriptor [53]. Incorporating a second, more electronegative metal such as Pd into a Cu‐based heterostructure similarly reshapes the local electronic structure at the Cu−Pd interface, illustrating that d‐band engineering through bimetallic contact is a transferable strategy that operates whether the host architecture is a discrete alloy nanoparticle, a heterostructure, or a MOF node [54]. Within MOF and MOF‐derived architectures specifically, the Cu−Co and Cu−Ni examples described above belong to a broader family of bimetallic nodes engineered for synergistic adsorption and hydrogenation behavior. Synergistic integration of copper and cobalt within a single MOF node has been reported to combine the strong *NO_3_ affinity associated with Cu sites and the hydrogenation activity associated with Co sites, consistent with the d‐band rationale outlined for Cu_1_Co_1_HHTP [84]. MOF‐derived Cu/Co‐doped nanocarbons extend this synergistic adsorption–hydrogenation mechanism to pyrolyzed, carbon‐supported node derivatives, indicating that the electronic cooperation between Cu and Co centers persists after node transformation [136]. Analogous bimetallic pairings based on Fe and Ni, namely, CuFe‐ and NiFe‐MOF nodes, achieve dual‐functional behavior in which one metal preferentially activates nitrate while the other supplies active hydrogen, reinforcing Cu−Co and Cu−Ni as specific instances of a general Fe/Co/Ni/Cu bimetallic design strategy for NO_3_^−^RR [137, 138]. Beyond homogeneous alloying within a single node, deliberately engineered phase boundaries provide an additional route to redistribute electron density. Interfacial polarization at Cu/Pd/CuO_*x*_ multiphase heterostructures reconstructed from a parent MOF concentrates charge redistribution at the phase boundary itself, generating locally distinct electronic environments for nitrate activation and hydrogenation that are not accessible in a single homogeneous alloy phase [139]. MOF‐on‐MOF heterostructures, in which two chemically distinct frameworks are grown in intimate contact, achieve a related effect at a coarser length scale, using the interface between two crystalline MOF phases rather than a single bimetallic node to establish complementary active‐site environments [140]. These interfacial strategies indicate that d‐band engineering is not limited to substitutional doping of a single node but can also be achieved by controlling the spatial arrangement of chemically distinct phases at the nanoscale. A further CuNi‐MOF catalyst, designed specifically to lower the overpotential for nitrate‐to‐ammonia conversion, corroborates the PDS switch from NO_2_^−^ hydrogenation to *NO hydrogenation identified for Cu−Ni alloys and shows that this switch can be reproduced in a directly synthesized, rather than postsynthetically alloyed, bimetallic MOF node [141]. Taken together with the metal‐node identity effects and the secondary coordination sphere strategies, bimetallic and heterostructure engineering constitute a third, largely orthogonal design axis: whereas node identity and ligand functionalization primarily tune the local adsorption energetics at a single active site, d‐band engineering through alloying or interfacial contact redistributes electron density across multiple neighboring sites, allowing the RDS and PDS to be tuned concertedly rather than independently.

Collectively, these structure‐barrier relationships demonstrate that rational selection of metal nodes, ligand functional groups, and dual‐site arrangements allows independent or concerted tuning of the RDS and PDS. As a result, MOF‐based electrocatalysts can shift selectivity from the thermodynamically preferred N_2_ pathway **(Reaction 4)** toward the highly selective NH_3_ pathway **(Reaction 3)** [65, 86, 92, 93].

(9) $${\mathrm{NH}}_{2}\mathrm{OH}+2H^{+}+2e^{-}\;\to \;\mathrm{NH}3+H_{2}O\;E\hat{A}{}^{\circ}=0.42\text{V vs SHE}$$

### 2.4 Activity and Selectivity Descriptors

Typically, catalytic performance in NO_3_^−^RR is assessed by onset potential, current density, and FE with a focus on selectivity in favor of NH_3_ over competing byproducts [83]. The linear sweep voltammograms (LSV) of 0.1 M Na_2_SO_4_ with 50 mg L^–1^ NO_3_^–^ generally reflect very high cathodic currents compared to the same electrolyte without nitrate, which provides direct evidence for the presence of NO_3_^−^RR activity (Figure 2e) [83]. Activity descriptors are very sensitive to catalyst composition optimization. For instance, the optimum Co/Fe ratio for Cox@MIL‐Fe is found at Co_0.1_@MIL‐Fe, which has the highest ECSA as measured by a double‐layer capacitance (Cdl) of 2.55 mF cm^−2^ (Figure 2f) [83]. Similarly, Cu‐MOF‐74 preferentially catalyzes NO_3_^−^ to NO_2_^−^ **(Reaction 7)** conversion, and insufficient proton‐donating sites can lead to surface poisoning and reduced stability. However, the Cu@Co‐MOF‐74 exhibit superior catalytic activity from the Cu‐MOF‐74 and Co‐MOF‐74 due to its highest ECSA (0.4271 mF cm^−2^) (Figure 2g), while the electrochemical impedance spectroscopy (EIS) examined the charge transfer resistance (Rct) of the Cu@Co‐MOF‐74 composite, which is reduced to 128.3 Ω, as compared to Co‐MOF‐74 which is 191.7 Ω (Figure 2h) [84]. The activity trends are also understood by electronic structure descriptors based on DFT. In Cu1Co1HHTP, the incorporation of Co in the Cu structure moves the position of the d‐band closer to the Fermi level (−3.22 eV compared to −3.47 eV), which increases the adsorption of the key reaction intermediates and thus improves the NO_3_^−^RR kinetics [62]. The potential determining step (PDS) overpotential can be synergistically reduced in Fe–Co(OH)F/Co‐TEPB systems by the Fe sites having a low NO_3_^−^ adsorption energy and the Co sites providing the hydrogen supply [92]. Important activity and selectivity descriptors are therefore the d‐band center, determining *NO activation, surface coverage of *H, which influences the selectivity between hydrogenation and competing HER, and the adsorption energies of N‐containing intermediates, which determine product selectivity [86]. Experimentally, lower onset potentials in NO_2_^−^ containing electrolytes as compared to NO_3_^−^ systems suggest that stepwise reduction pathways are favored. The broad metal screening studies (Sn, Bi, Pb, Al, Zn, and In) also show that pH is effective in altering the reaction rates as well as the product distributions, which again confirms that the proton availability is a kinetic descriptor [84, 142]. Overall, synergistic bimetallic effects‐particularly the promotion of Cu or Fe active sites by Co‐emerge as a central design principle. These interactions increase activity by fine tuning the electronic structure, optimizing the intermediate adsorption, and inhibiting parasitic reactions, all of which contribute to higher NH_3_ selectivity and NO_3_^−^RR efficiency [62, 83, 84].

## 3 MOFs as Electrocatalyst Platforms

Because of its strong affinity toward NO_3_^−^, copper (Cu) has attracted considerable attention in the rapidly expanding field of NO_3_RR research, which aims to develop highly efficient electrocatalysts with high FE and NH_3_ yield rate. Nevertheless, at low overpotentials, NO_3_^−^RR has a poor FE (<50%), resulting in low energy efficiency. Furthermore, the systematic design of NO_3_^−^RR is necessary not only to evaluate its economic viability but also to provide data beyond H‐type cells since the NH_3_ yield is still not comparable to that of the industrial Haber–Bosch process [143]. MOFs and their derivatives are thought to be potential electrocatalysts in the field of nitrogen electrochemistry for crucial activities including ammonia production and nitrogen conversion [144]. An MOF is a crystalline porous material having a periodic network structure [145]. It is constructed through the self‐assembly of inorganic metal centers (metal ions or metal clusters) and bridging organic ligands [146]. Short diffusion lengths for charge carriers can be achieved during the electrocatalytic reaction because the metal centers in MOFs separated by organic linkers can be regarded as quantum dots. By adjusting the organic ligands and/or metal centers, the surface area and band gap of MOFs can be tuned to optimize their electrocatalytic activity [147]. MOF have attracted extensive interest as efficient electrocatalytic materials because of their large surface area, diverse architectures, tunable porosity, and uniformly distributed active sites arising from unsaturated metal centers [148]. Their highly ordered pore structures and adjustable pore sizes enable molecular‐level size selectivity. In addition, interfacial interactions between the metal core and the MOF shell can modify the electronic structure of the catalytic active sites [149]**.** For example, Wang et al. [128] used the in situ development of a Zr‐based MOF (UiO‐66) around a CuZn nanocluster (UiO‐CuZn) to construct a confined architecture with molecular‐level channels. In this system, the MOF framework serves as a proton‐relay network through coordination between the CuZn nanocluster and the Bronsted acidic groups inside the pores. A low‐barrier hydrogen‐bonding channel is formed by the ordered arrangement of acidic nodes, which accelerates proton transport to the active sites. This is crucial for the highly proton‐demanding NO_3_^−^RR (8e/9H process) as it prevents proton depletion at high current densities, which often causes poor selectivity in unconfined catalysts. In addition, the MOF shell can prolong the catalyst lifetime by protecting the active metal core from poisoning by complex species in real wastewater matrices. A CoP/Zn‐ZIP composite was reported to maintain an ammonia production rate of 0.86 mmol cm^−2^ h^−1^ and a FE of 95% following 30 consecutive cycles of electrolysis [150]. However, in general, the direct use of MOFs as electrocatalysts is hindered by their poor electrical conductivity and instability in electrochemical environments. Designing effective electrocatalysts based on MOFs involves careful consideration of both electrical conductivity and the presence of redox‐active metal centers, as these factors are pivotal for catalyzing electrochemical reactions [151].

For example, MOF can serve as catalyst templates or precursors for the preparation of functionalized MOF derivatives via high‐temperature pyrolysis, improving electrocatalytic performance. This approach is also effective for producing high‐quality electrocatalytic materials [114]. This approach has several distinct advantages: (1) It does not require the addition of chemicals and hence spares the production of secondary pollutants; (2) it allows precise control of the product, especially valuable NH_3_, with rational catalyst design and optimization of operating parameters; and (3) it has a small footprint because of its modular design and can easily and directly be operated with renewable electricity (e.g., solar and wind energy), making it a carbon neutral or even carbon‐negative water treatment process [121]. However, MOFs are still hindered by several drawbacks in the field of electrocatalysis; they possess low intrinsic activity, low electrical conductivity, low accessible actives, and low stability in the operation [148]. This approach has several distinct benefits: (1) No chemical additives are required, resulting in the elimination of secondary pollution; (2) the small footprint of the modular reactor allows rational design and optimization of the catalyst of the operating parameters, which enables control of the product formation, in particular, towards valuable NH_3_; (3) the direct usage of renewable electricity (e.g., solar or wind) power the modular reactor, opening the way to carbon‐neutral or even carbon‐negative water treatment processes [152]. Although MOFs have some of these benefits, they are still plagued by several drawbacks in electrocatalysis, such as low activity, poor conductivity, low accessible active surface, and poor stability under operating conditions. To address these challenges, several strategies have been proposed, including the construction of two‐dimensional MOF nanosheets or hierarchical nanostructures, the incorporation of secondary metals into monometallic MOFs, and the formation of with other materials [65]. Although growing MOF materials on nanoparticles (NPs) can provide integration and protection for the NPs, diffusion limitations may arise between the MOF surface and NPs. Although loading NPs onto or into MOF materials ensures contact between the NPs and reactants and allows fine control over physical and electrochemical properties, it may also cause pore blockage in the MOFs and stability problems for the NPs. Although simultaneous formation of both components simplifies the synthesis process and promotes uniform distribution of NPs within MOF particles, it may introduce defects and offer limited control over the individual components [144].

### 3.1 Dynamic Active Sites

Although MOF metal nodes are frequently treated as static structural units, under the electrochemical bias of nitrate reduction, they commonly undergo dynamic reconstruction, valence‐state evolution, and the reversible generation of defective active sites [122, 139]. These processes are central to activity and selectivity yet have received only limited systematic attention in the MOF–NO_3_RR literature. Under the potential window of NO_3_^–^RR, the Pourbaix lines of first‐row transition‐metal couples (Cu^2+^/Cu^+^, Fe^3+^/Fe^2+^) frequently lie within or near the operating range. Consequently, partial reduction of the metal nodes occurs during electrolysis, altering both the adsorption energy of NO_3_^–^ and the subsequent hydrogenation barriers [65, 89]. This valence‐state evolution constitutes a dynamic handle that continuously modulates the electronic structure of the active sites. In flexible bimetallic frameworks, lattice distortion arising from pseudo‐Jahn–Teller effects can further redistribute d‐orbital electrons and shift the d‐band center, optimizing intermediate binding [132]. A clear illustration of node reconstruction is provided by CuPd‐MOF precatalysts. Under operando nitrate reduction conditions, the parent framework is reconstituted into Cu/Pd/CuO_*x*_ multiphase heterostructures (Figure 3a,b). The newly formed interfaces induce interfacial polarization and electron transfer from Pd to Cu, creating electron‐deficient Pd sites that preferentially adsorb NO_3_^–^ and electron‐rich Cu sites that facilitate subsequent hydrogenation steps [139]. The reconstruction therefore converts a molecularly defined MOF into a multiphase catalyst whose active sites are generated in situ. More direct evidence for dynamic active‐site evolution has been obtained by operando X‐ray absorption spectroscopy (XAS). In Cu species derived from MOFs@CuPc precursors, the restructuring behavior among single‐atom, cluster, and nanoparticle Cu sites is codependent on both Cu loading and applied potential. Operando XAS reveals an incompletely reversible transformation: the restructured CuN_4_&Cu_4_ ensembles that form under cathodic bias deliver the highest NH_3_ yield (88.2 mmol h^–1^ g_cat_^–1^) and FE (~94.3%) at –0.75 V versus RHE owing to optimized NO_3_^–^ adsorption and accelerated conversion of the *NH_2_OH intermediate (Figure 3c–e) [122]. The partial reversibility of the structural change upon return to open‐circuit potential underscores that the catalytically competent sites are dynamic rather than static. Complementary operando Raman and FTIR studies further capture the evolution of both surface intermediates and the local coordination environment. On ligand‐engineered Cu_3_‐MOFs, potential‐dependent Raman bands track the sequential formation of *NO_2_, *NO, and *NH_2_OH while simultaneously reflecting changes at the metal centers [112]. Intentional introduction of coordination defects in Cu‐BTC frameworks increases the density of under‐coordinated Cu sites, thereby elevating the electrochemically active surface area and accelerating the multielectron cascade to NH_3_ [133]. Collectively, these spectroscopic observations demonstrate that defective active sites can be generated reversibly under operando conditions and that their formation is intimately linked to the applied potential and local chemical environment.

**FIGURE 3:**
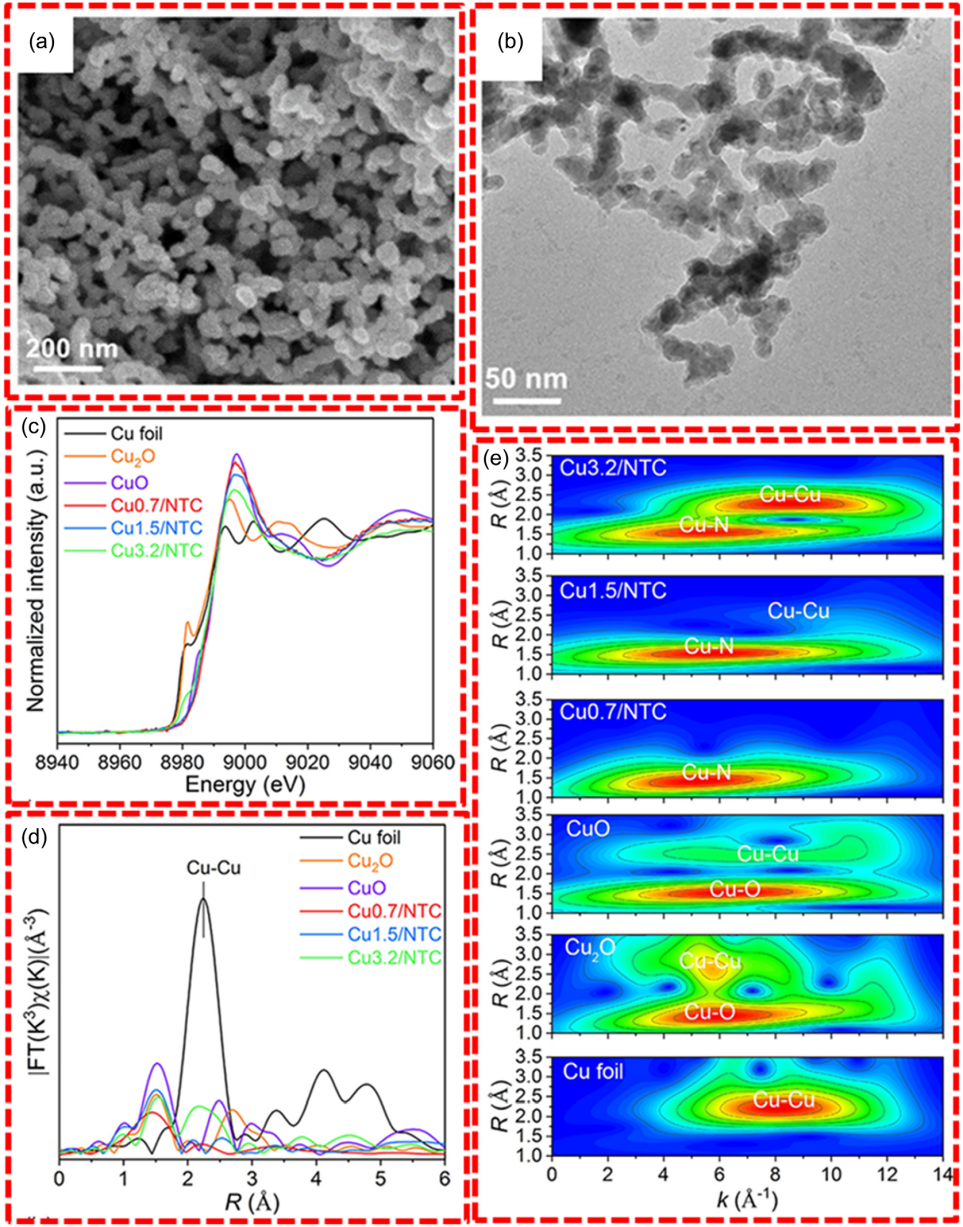
(a) SEM image and (b) TEM image of the postreconstructed CuPd‐MOF (i.e., Cu/Pd/CuO_*x*_). Reproduced with permission from [139]. Copyright 2022, Elsevier. (c) Cu K‐edge X‐ray adsorption near‐edge structure (XANES), (d) k^3^‐weighted Fourier‐transformed extended X‐ray absorption fine structure (FT‐EXAFS), and (e) wavelet transform extended X‐ray absorption fine‐structure (WT‐EXAFS) spectra of Cu foil, Cu_2_O, CuO, Cu0.7/NTC, Cu1.5/NTC, and Cu3.2/NTC. Figure reproduced from [122], which is open access and permits unrestricted use of materials under the term of the Creative Common license CC‐BY.

In short words, the dynamic character of MOF‐derived active sites encompassing metal‐node reconstruction, valence‐state evolution, and reversible defect generation must be regarded as an intrinsic feature of NO_3_^–^RR catalysis rather than an incidental phenomenon [122, 139]. Systematic operando characterization (XAS, Raman, and FTIR) is therefore indispensable for correlating structural dynamics with catalytic performance and for guiding the rational design of next‐generation MOF‐based electrocatalysts.

## 4 MOF‐Based Electrocatalysts for NO_3_^–^RR

### 4.1 Reaction Mechanism and Selectivity Control

#### 4.1.1 Nitrate Adsorption and Activation on Metal Centers

The electrocatalytic NO_3_^−^RR commences with the adsorption of NO_3_^−^ onto catalytic active sites, a process enabled by the substantially lower bond energy of the N═O bonds in NO_3_^–^ relative to the N^–^═N bond in N_2_ [153]. This adsorption step serves as the prerequisite for further reduction and is highly governed by the electronic configuration of the metal centers. As studied by Mao and colleague [116] on Fe−Co bimetallic catalysts derived from MOF‐74, the free‐energy variation associated with *NO_3_ generation form NO_3_^−^ is −1.46 eV on the bimetallic surface, comparable to that observed on monometallic Fe (−1.54 eV) but significantly more positive than that on Co (−2.01 eV), as illustrated in Figure 4a. A higher NO_3_^–^ binding at Co sites is favorable for initiating adsorption but may be disadvantageous for subsequent desorption processes; incorporation of Fe into the Co lattice induces a pronounced redistribution of electron density, as revealed by differential charge‐density analysis (Figure 4b), which improves nitrate activation. Electron density accumulates on the adsorbed nitrate while depleting from the Fe center, corresponding to directional electron transfer from Fe to *NO_3_ [116]. This directional charge transfer injects electron density into the π* antibonding orbitals of the N−O bonds, thereby elongating and weakening those bonds and facilitating their subsequent cleavage [124, 136]. The extended Fe−O bond length relative to Co−O also favors the dissociation of the N−O bond, which increases the overall NO_3_^−^RR kinetics [116]. Analogous orbital‐matching arguments explain the efficacy of Cu‐based centers, whose highly occupied d orbitals align energetically with the lowest unoccupied π* orbital of NO_3_^–^, enabling efficient charge injection and N–O bond activation [136]. Collectively, these electronic structure effects convert the initial adsorption step into a thermodynamically and kinetically favorable entry point for the multielectron cascade. In another study, Zhang et al. [124] synthesized a cobalt‐doped Fe@Fe_2_O_3_ catalyst from Fe‐MOF‐74, which lowers the d‐band center of adjacent iron sites, enhances the orbital overlap, and facilitates the direction of charge transfer from nitrate to the metal d‐orbitals, thereby strengthening the adsorption of nitrate and simultaneously weakening N─O bonds, which makes the Fe−Co ensembles the primary active sites for nitrate capture and later activation for reduction. In the meantime, cobalt dopants increase the intermediate adsorption energies and suppress competing HER, which can direct the pathway toward ammonia for efficient reduction. Similarly, in Co_1_Cu_2_/NC/Ni, NO_3_^−^ preferentially adsorbs on Cu at high Cu d‐orbital electron occupancy, and then electron transfer occurs from the electron‐rich Cu_2_O interfaces to the π* antibonding orbital of the adsorbed NO_3_^−^, producing elongation of the N─O bonds and their rupture [136]. This pathway is further tuned by structural effects in Cu−Ni alloys: When Ni atoms are incorporated in the structure, the adsorption of NO_2_* changes from two neighboring Cu atoms (monometallic Cu) to mixed Cu−Ni or Ni−Ni sites, which lowers the energy barrier to the following hydrogenation steps, as illustrated in Figure 4c [135]. NO_3_^−^ and NO_2_^−^ can be selectively chemisorbed on the N‐doped carbon shell and then be reduced by the FeN core, while the in situ formed H_2_ is used as a reductant and a protecting agent for FeO from oxidation, as shown in Figure 4d [154].

**FIGURE 4:**
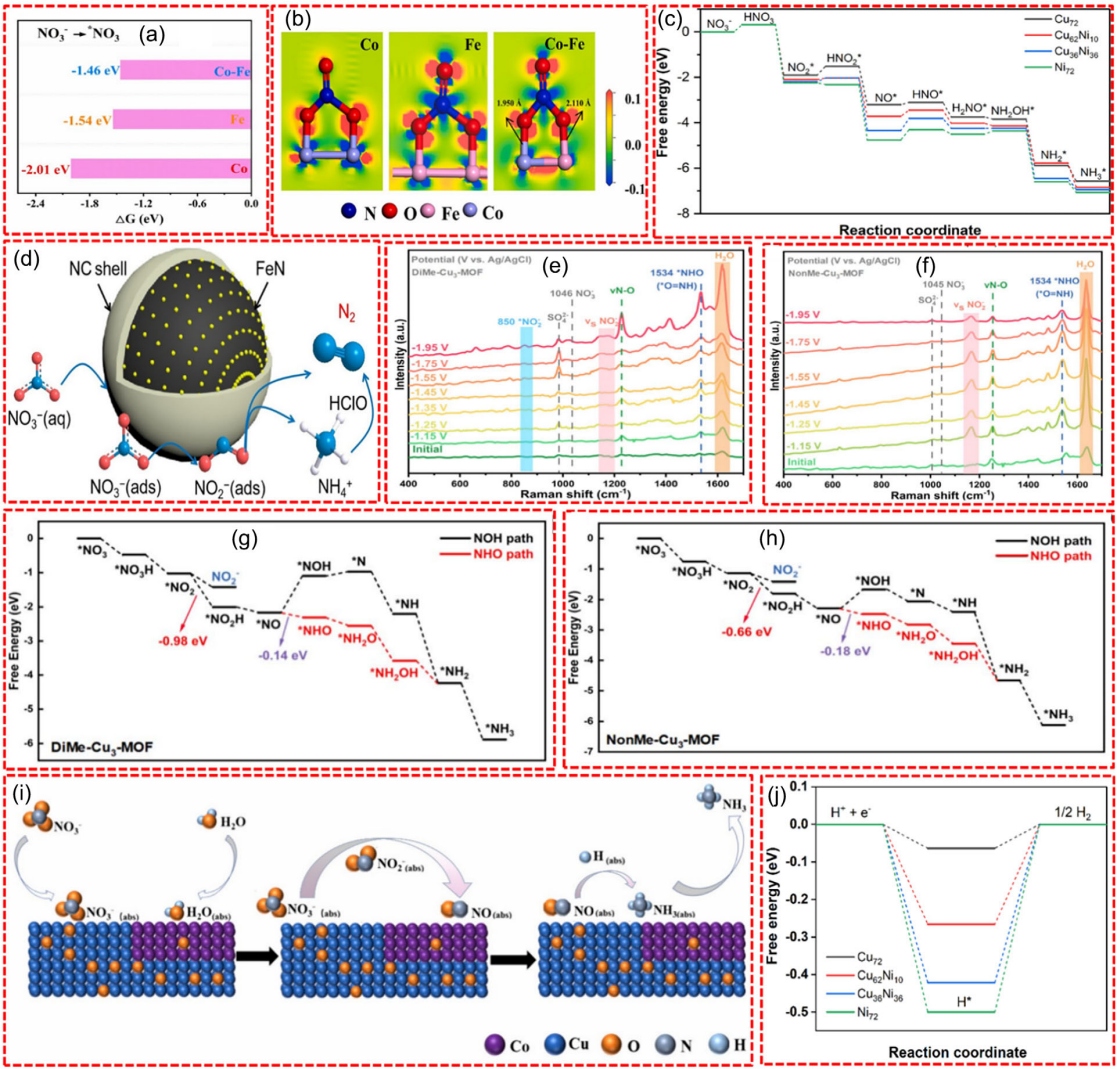
(a) Free energy change for the conversion of NO_3_^−^ to *NO_3_, (b) charge density differences. Reproduced with permission from [116]. Copyright 2025, Elsevier. (c) Reaction free energy profile on Cu_72_, Cu_62_Ni_10_, Cu_36_Ni_36_ and Ni_72_ surfaces. Reproduced with permission from [135]. Copyright 2022, Elsevier. (d) Schematic illustration of the nitrate reduction reaction (NO_3_^−^RR) mechanism over FeN‐NC. Reproduced with permission from [154]. Copyright 2020, Elsevier. (e) Operando Raman spectra of DiMe‐Cu_3_‐MOF recorded at different applied potentials in 50 mM KNO_3_ and 0.5 M K_2_SO_4_ electrolytes, (f) operando Raman spectra of NonMe‐Cu_3_‐MOF recorded under identical condition, (g) Gibbs free energy diagram for the NO_3_^−^RR on DiMe‐Cu3‐MOF, and (h) Gibbs free energy diagram for the NO_3_RR on Non‐Me‐Cu_3_‐MOF. Figure reproduced from [112], which is open access and permits unrestricted use of materials under the term of the Creative Common license CC‐BY. (i) Proposed electrocatalytic nitrate reduction mechanism over the Co_1_Cu_2_/NC/Ni. Reproduced with permission from [136]. Copyright 2026, Elsevier. (j) Gibbs free energies of hydrogen adsorption and H_2_ formation on Cu_72_, Cu_62_Ni_10_, Cu_36_Ni_36_ and Ni_72_ surfaces. Reproduced with permission from [135]. Copyright 2022, Elsevier.

#### 4.1.2 Stabilization of Key Intermediates

The stabilization of key reaction intermediates is critical to ensure that the multielectron/proton transfer sequence leads to the formation of ammonia, rather than competing reaction paths. Fu et al. [112] demonstrated that in situ Raman and FTIR analyses, together with DFT calculations, reveal that the NHO pathway (*NO_3_^−^ → *NO_2_ → *NO_2_H → *NO → *NH_3_) dominates on Cu_3_‐MOF‐derived catalysts, with the transformation from *NO to *NOH recognized as the RDS, as indicated in Figure 4e,f. On the DiMe‐Cu_3_‐MOF, hydrogen bond interactions between methyl‐functionalized ligands and *NO_2_ reduce the Gibbs free energy for *NO_2_ → *NO_2_H conversion to −0.98 eV, compared with −0.66 eV on the NonMe‐Cu_3_‐MOF, thereby thermodynamically favoring ammonia production, as illustrated in Figure 4g,h. The covalent interaction analysis is useful in confirming strong van der Waals interactions between methyl groups and reaction intermediates that provide extra stabilization that has been experimentally confirmed. This specially designed microenvironment simultaneously raises the free‐energy barrier for the HER to enhance NO_3_^−^RR selectivity. Ren et al. [139] revealed this phenomenon in an in situ reconstructed Cu/Pd/CuO_*x*_ multiphase heterostructure obtained from a CuPd‐MOF. Charge transfer from Pd toward Cu across heterogeneous interfaces produces electron‐deficient Pd regions and electron‐rich Cu regions. This means that the electron‐deficient Pd centers can specifically bind and activate nitrate and catalyze its first hydrogenation to NO_2_^−^, with subsequent nitrogen‐containing intermediates, while the electron‐rich Cu centers catalyze the subsequent hydrogenation steps. The polarized Pd sites, on the other hand, modulate the availability of hydrogen, thus controlling the formation of N_2_ and promoting high selectivity toward ammonia. For Cu–Ni alloys, the increase in potential of the PDS from NO_2_ hydrogenation (0.39 eV on Cu) to NO* hydrogenation (0.27 eV on optimal Cu_62_Ni_10_) reduces accumulation and release of toxic NO_2_^−^ and allows efficient conversion to NH_3_ [135].

#### 4.1.3 Multielectron Transfer at Single Versus Multinuclear Sites

The nuclearity and spatial arrangement of metal‐active sites are important factors governing multielectron transfer and product selectivity during NO_3_^–^RR. Mononuclear or isolated metal sites provide well‐defined coordination and electronic environments that can be particularly effective for NO_3_^–^ adsorption, electron transfer, and initial N─O bond activation as demonstrated for isolated FeN centers and atomically precise Co‐MONTs Mononuclear or isolated metal sites provide well‐defined coordination and electronic environments that can be particularly effective for NO_3_^–^ adsorption, electron transfer, and initial N─O bond activation as demonstrated for isolated FeN centers and atomically precise Co‐MONTs [154, 155]. In contrast, multinuclear and bimetallic architectures can introduce additional metal–metal interactions and cooperative catalytic functions, allowing neighboring centers to participate in nitrate activation, water dissociation, proton/active hydrogen supply, and subsequent hydrogenation of nitrogen‐containing intermediates [83, 116, 135, 136]. Thus, the distinction is not that single‐metal sites are restricted exclusively to NO_3_^–^activation but rather that multinuclear architecture provides additional degrees of freedom for coordinating the different elementary steps of NO_3_^–^ → NO_2_^–^ → *NO → NH_3_ pathway. In isolated site systems, the electronic structure and coordination environment of the individual metal center strongly influence nitrate adsorption and activation [156]. For example, the isolated FeN center reported for FeN–NC–140 acts as a strong electron‐donating center, while the surrounding N‐doped carbon environment participates in electron transport and nitrate conversion [154]. Similarly, atomically precise Co‐MONTs provide a well‐defined coordination environment in which the Co centers and coordinated water molecules generate highly positive electrostatic potential regions, strengthening the interaction between Co 3d and NO_3_^–^ 2p orbitals and lowering the energy barrier for nitrate activation [155]. These examples highlight the importance of the isolated active site and nitrate during the initial stages of NO_3_^–^RR. Multinuclear configurations, however, can provide cooperative functionality by electronically and geometrically coupling neighboring metal centers. In Fe–Co and Co–Cu systems, neighboring metals modify charge distribution and electronic structure while providing complementary functions for nitrate activation and subsequent hydrogenation [136, 157, 158]. In the Co_1_Cu_2_/NC/Ni system, nitrate preferentially adsorbs on Cu sites, whereas neighboring Co sites contribute to H_2_O activation and proton supply, thereby establishing a cooperative environment for the subsequent reduction of nitrogen‐containing intermediates (Figure 4i) [136]. Likewise, optimized Cu_62_Ni_10_ alloying modifies the adsorption configuration of key intermediates and suppresses the competing HER (Δ*G*_H_ = 0.27 eV compared with 0.06 eV on Cu) (Figure 4j), thereby promoting the complete eight‐electron sequence to NH_3_ with high selectivity and reducing toxic NO_2_^−^ release [135]. These examples demonstrate that the principal advantage of multinuclear architecture arises from the cooperative coupling of catalytic functions rather than simply from an increased number of metal centers. The cooperative role of multinuclear active sites can be further extended from bimetallic pairs to well‐defined polynuclear metal clusters, where geometric symmetry and nanoconfined environments provide additional control over charge transfer and hydrogenation pathways. For instance, Wang et al. developed high‐symmetry coordination metal organic polymers (MOPs, e.g., Co‐TATB) featuring square‐planar tetranuclear cluster building units. This structural configuration facilitates reactant and electron transfer and favors the adsorption of active hydrogen (*H), which is important for the proton‐demanding NO_3_^−^ reduction process. Furthermore, the highly positive electrostatic potential at the Co sites promotes nitrate interaction and rapid interfacial charge transfer, resulting in a FE of approximately 98% [114]. Similarly, polynuclear cobalt‐cluster coordination polymers such as NJUZ‐2 and NJUZ‐3 provide well‐defined porous and nanoconfined environments that facilitate NO_3_^−^ adsorption and reduce the energetic requirements for hydrogenation of *NO_3_ and *NO_2_ intermediates. In a flow‐cell configuration, the catalyst achieved a current density of 469.9 mA cm^−2^ and NH_3_ yield rate of 3370.6 mmol h^−1^ g^−1^ [117]. The structure function relationship between mononuclear and multinuclear active sites in MOF‐based NO_3_^−^RR is systematically summarized in Table 1.

**TABLE 1:** Structure function relationship of mononuclear and multinuclear active sites.

Active‐site configuration	Representative examples	Predominant catalytic role	Key functions	Advantages
Mononuclear/isolated single metal site	FeN center [154] Co‐MONT [155].	Nitrate adsorption and initial activation	NO_3_^−^ adsorption, electron transfer, and initial N–O bond activation	Well‐defined coordination and electronic environment
Bimetallic/multinuclear site	Fe–Co [83, 116] Cu–Co [136] Cu–Ni [135].	Cooperative nitrate activation and sequential hydrogenation	Cooperative charge redistribution, H_2_O activation, proton/active‐H supply, and intermediate hydrogenation	Functional cooperation between neighboring metal centers
Polynuclear metal‐cluster site	Co‐TATB [114] NJUZ‐2/NJUZ‐3 [117]	Integrated multistep NO_3_^−^RR	Nanoconfined charge transfer, *H regulation, nitrate activation, and hydrogenation of N‐containing intermediates	Multiple coupled sites with spatially organized reaction pathways

#### 4.1.4 Comparative Analysis of Charge‐Transfer Pathways in MOF‐Based Electrocatalysts

Three principal charge‐transport channels can be identified and compared on a common electronic‐structure basis. **(1)** Metal‐node–organic‐linker channels operate through coordination‐sphere effects and secondary interactions (e.g., hydrogen bonding between functionalized ligands and *NO_2_), stabilizing key intermediates without long‐range electron delocalization [112]. **(2)** Bimetallic heterointerfaces (Fe–Co, Cu–Co, Cu–Ni, and Cu–Pd) generate interfacial polarization and directional electron transfer. Differential charge‐density analyses consistently show electron flow from one metal center toward the adsorbed nitrate, populating N─O antibonding orbitals, elongating metal–oxygen contacts, and shifting the d‐band center closer to the Fermi level. These rearrangements simultaneously facilitate N─O cleavage (RDS) and stabilize NOH/HNO (potential‐determining step) while suppressing the HER [116, 124, 135, 136, 139]. **(3)** MOF–carbon support interfaces (N‐doped carbon shells, FeN–NC, and Co_1_Cu_2_/NC) provide continuous electron‐conduction pathways and additional adsorption sites. The carbon matrix delivers electrons rapidly to the metal centers and can itself chemisorb NO_3_^−^/NO_2_^−^, while the metal core executes the multielectron reduction [136, 154]. The common mechanistic feature uniting these three channels is directional electron transfer that populates antibonding orbitals of nitrogen‐oxo intermediates, weakens N─O bonds, and thereby lowers both kinetic and thermodynamic barriers of the eight‐electron pathway to NH_3_. Rational design of MOF‐based electrocatalysts therefore requires deliberate balancing of the relative contributions of these three pathways under the operating conditions.

### 4.2 Applications of MOFs and MOF‐Based Materials for Electrocatalytic NO_3_^−^RR

The NO_3_^−^RR has recently emerged as a compelling strategy for addressing NO_3_^−^‐contaminated water while enabling value‐added ammonia synthesis under ambient conditions. In this context, MOFs and their derived architecture have attracted growing attention as adaptable electrocatalytic platforms owing to their structural periodicity, high‐surface areas, and precisely tunable coordination environments. The electronic structures, pore architecture, and active‐site distributions of these modular assemblies of metal nodes and organic linkers are rationally controlled and imperative for regulating multistep proton‐electron transfer pathways for NO_3_^−^ conversion. Furthermore, the single atomic centers of MOF‐based systems can be stabilized, bimetallic motifs can be built, and conductive carbonaceous derivatives can be obtained, which helps to overcome the intrinsic limits of conductivity of pristine MOFs. The combination of these properties makes MOFs and MOF‐derived materials promising candidates for improving activity, selectivity, and mechanistic control in electrolytic NO_3_^−^RR, which underpin their ever‐growing utilization in sustainable management of the nitrogen cycle and environmental remediation. Furthermore, Table 2 summarizes previous reported on the MOF‐based electrocatalyst for NO_3_^−^ reduction.

**TABLE 2:** Previous reported work along with performance metrices.

Catalyst	Active metal site(s)	Structural engineering strategy	**Electrolyte,** **mM**	pH	Applied potential	**NH**_**3**_ **yield rate**	FE, %	Nitrate conversion, %	**NH**_**3**_ **selectivity, %**	References
Fe_2_Co‐MOF	Trinuclear Fe_2_Co cluster	Trinuclear cluster‐based MOFs (Fe_2_M)	H_2_SO_4_ (500)	1	−1.1 V vs. RHE	20,653.5 μg h^−1^ mg^−1^	90.55	—	98.5	[159]
DiMe‐Cu_3_‐MOF	Trinuclear Cu(I) (Cu_3_) cluster	Hydrogen‐bond microenvironment engineering	KOH (100)	≈ 6.5	−1.55 V vs. RHE	401 μg cm^−2^ h^−1^	95	—	—	[112]
CuPd‐MOF	Cu, Pd, and CuOx interfacial active sites	Pd‐incorporated Cu‐MOF into a Cu/Pd/CuOx multiphase heterostructure,	K_2_SO_4_ (500)	Not fixed; changes during electrolysis	−1.30 V vs. SCE	1510.3 μg h^−1^ mg^−1^cat	84.04	86.81	90.06	[139]
Ir NCs@Co‐MOF	Ir nanoclusters (primary active sites) with Co sites	Embedding ultrasmall (~1.8 nm) p‐MBA ligand‐protected Ir nanoclusters into 2D Co‐MOF nanosheets	Na_2_SO_4_ (100)	—	−0.80 V vs. RHE	230.1 μg h^−1^ cm^−2^	—	92.5	81.4	[119]
Ru_*x*_O_γ_/Ni‐MOF (RuNi‐MOF)	Ru clusters (primary active sites	Anchoring Ru clusters onto a Ni‐MOF	Na_2_SO_4_ (100)	~ 7.0	−1.70 V vs. Ag/AgCl	2 mg h^−1^ mg cat ^−1^	73	—	Nearly 100	[120]
NiFe‐MOF nanosheet array	Synergistic Ni–Fe bimetallic active sites	Interface and morphology engineering	KOH (1000)	~ 14	−0.60 V vs. RHE	1.13 mmol h^−1^ cm^−2^	92	—	High	[138]
Fe@MOF‐808‐P	Fe active site	Phosphate (H_2_PO_4_^−^/HPO_4_^2−^) functionalization of Fe@MOF‐808.	K_2_SO_4_ (100)	7.0	−0.90 V vs. RHE	420.4 ± 18.1 μmol h^−1^ cm^−2^	90.8	—	—	[125]
MOF‐derived Cu/CeO_2_–C	Cu active sites	Support engineering via MOF derivation.	NO_3_^−^ aqueous electrolyte (500 mM)	7.0	−1.29 V vs. SHE	NH_3_ production was quantified mainly through TOF and selectivity.	62	—	73.4	[160]
Cu@Co‐MOF‐74	Cu and Co dual active sites	Bimetallic MOF engineering.	Na_2_SO_4_ (100)	7.0	−0.88 V vs. RHE	307.98 mmol h^−1^ g	84.01	—	100	[84]
MOF‐derived Cu_1_Co_5_–N–C	Cu–N_*x*_ sites and Co–N_*x*_/Co‐based sites	MOF‐derived tandem catalyst engineering	Na_2_SO_4_ (100)	—	−1.0 V vs. RHE	22.3 mg h^−1^ mg	97.53	—	—	[134]
Pd‐NDs/Zr‐MOF	Ultrafine Pd nanodots (Pd NDs) anchored on redox‐active Zr‐MOF	Interfacial reduction nucleation strategy	Na_2_SO_4_ (100)	—	—	287.31 mmol h^−1^ g^−1^	58.1	92	—	[121]
SO_3_‐MOF‐808‐coated Cu electrode	Cu active sites	Porous MOF coating engineering.	Na_2_SO_4_ (500)	7.0	−1.19 V vs. SHE	0.383 mmol/h^−1^ mg_cat_	87.5	—	95.6	[70]
MOF‐supported single‐atom Cu precatalyst	In situ formed Cu nanoclusters	Anti‐collapse MOF + single‐atom precursor engineering	Na_2_SO_4_ (500)	7.0	−0.9 V vs. RHE	66 μmol h^−1^ cm^−2^	85.5	—	93.3	[123]
Co‐TEPB/Fe–Co (OH)F	interfacial Fe–Co dual active sites	MOF‐on‐metal hydroxide heterostructure with interfacial dual active sites	KOH (1000)			22.45 ± 0.48 mg h^−1^ cm^−2^	99.63	—	—	[161]
MOF‐derived FeCoS_2_/Fe_0.95_S_1.05_ bimetallic sulfide	Synergistic Fe–Co dual active sites	MOF‐derived bimetallic sulfide heterostructure	—	Alkaline	−0.8 V vs. RHE	2.705 mg h^−1^ mg	94.79	—	96.88	[116]
MOF‐derived CuNi/NC	Synergistic Cu–Ni alloy active sites	Pyrolysis of bimetallic MOFs		7.0	−0.8 V vs. RHE	≈ 0.73 mmol h^−1^ mg	79.6	—	94.4	[135]
Hierarchical porous Bimetallic CuFe(1:2) MOF	Synergistic Cu–Fe dual metal sites	Bimetallic MOF engineering + hierarchical porous nanostructure.	Na_2_SO_4_ (100)	—	—	5.08 mmol h^−1^ cm^−2^	87	High	—	[115]
Fe–Co (OH)F/Co‐TEPB	Fe, Co	MOF‐on‐metal hydroxide heterostructure	KOH (100)	Alkaline medium	—	22.45 mg h^−1^ cm^−2^	99.63	—	High	[92]
Co–N–C‐500	Coordination‐unsaturated Co nanoparticles	MOF‐derived low‐crystallinity engineering	K_2_SO_4_ (50)	7.0	−0.60 V vs. RHE	1.14 mg h^−1^ cm^−2^	78.6	—	—	[126]
Co‐HHTP/Ni‐BDC	Interfacial Ni and Co dual active sites	MOF‐on‐MOF heterostructure engineering	KOH (100)(100)100	≈14	−0.70 V vs. RHE	11.46 mg h^−1^ cm^−2^	98.4	—	—	[162]
Co–NC	Metallic Co NPs embedded within N‐doped graphitic carbon layers	MOF‐derived carbon engineering	KOH (100)	Strongly alkaline	−0.50 V vs. RHE	1.96 mg h^−1^ cm^−2^	35.01	—	—	[127]
CuNi‐TBC‐C	Cu and Ni dual active sites	Bimetallic Cu–Ni catalyst engineering	KOH (1000)	Strongly alkaline	−0.10 V vs. RHE	214 μmol h^−1^ cm^−2^	94.68	—	—	[141]
Cu/Cu_2_O@C	Cu^0^/Cu^+^ (Cu and Cu_2_O) active sites	MOF‐derived heterostructure engineering.	KOH (100)	Alkaline	−0.60 V vs. RHE	158.66 μmol cm^−2^ h^−1^	76.97	—	—	[163]
agCu(MeIm)_2_‐CC	Low‐coordination Cu (111) nanoparticles	MOF glass engineering + binder‐free self‐supporting electrode	KOH (1000)	—	−1.80 V vs. Ag/AgCl (sat. KCl)	Absolute value not reported	91	—	—	[164]

Gao et al. created a MOF‐on‐metal‐hydroxide hetero‐structured catalyst by wrapping the Fe–Co(OH)F nanowire with Co‐TEPB MOF nanosheets, which allows for creating multiple active sites for nitrate reduction (Figure 5a). The Fe centers can adsorb and activate NO_3_^−^, and the Co sites adjacent to Fe can facilitate the decomposition of H_2_O to produce surface hydrogen for intermediate hydrogenation. This synergistic mechanism reduces the energy barrier of the NO_3_^−^RR, resulting in an outstanding performance of NH_3_ production rate (22.45 ± 0.48 mg h^−1^ cm^−2^) and FE (99.63 ± 0.34%), which are higher than most other reported MOF‐based electrocatalysts (Figure 5b). The outcome of these results gives insights to the design of high‐performance nitrate‐to‐ammonia electrocatalysts [92].

**FIGURE 5:**
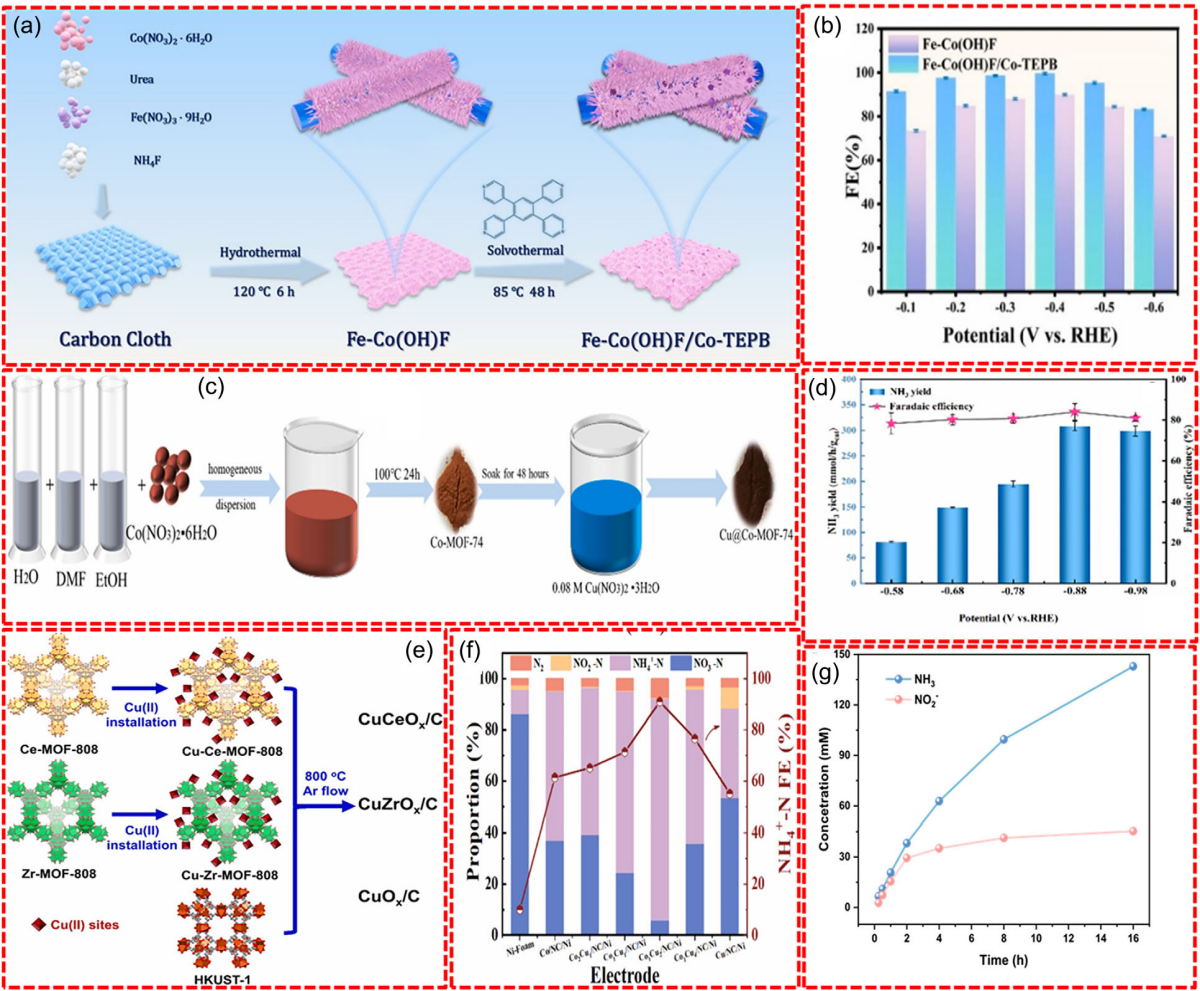
(a) Illustration of catalyst fabrication procedures, (b) FEs for NH_3_ generation via NO_3_^−^RR on Fe–Co(OH)F and Fe–Co(OH)F/Co‐TEPB across applied potentials from −0.1 to −0.6 V versus RHE. Reproduced with permission from [92]. Copyright 2025, Elsevier. (c) Schematic representation of the synthesis route for Cu@Co‐MOF‐74, (d) Comparison of NH_3_ production rates and FE (NH_3_) for Cu@Co‐MOF‐74 at various applied potentials (*t* = 3600 s). Reproduced with permission from [84]. Copyright 2025, Elsevier. (e) Preparation strategy of MOF‐derived Cu electrocatalysts supported on CeO_2_/carbon, ZrO_2_/carbon, and carbon substrates. Reproduced with permission from [160]. Copyright 2025, Elsevier. (f) Product distribution and NH_4_^+^‐N FE after 90 min electrolysis. Reaction conditions: [Na_2_SO_4_] = 0.1 mol L^−1^, [NO_3_‐N] = 100 mg L^−1^, applied potential = −1.5 V versus Ag/AgCl, pH = 7. Reproduced with permission from [136]. Copyright 2026, Elsevier. (g) Temporal concentration profiles of NH_3_ and NO_2_^−^ during potentiostatic electrolysis at −0.9 V versus RHE; corresponding electrolysis at −0.9 v versus RHE; corresponding FE(NH_3_) and NH_3_ yields of Fe@MOF‐88‐p at different potentials. Reproduced with permission from [125]. Copyright 2026, Elsevier.

Xin et al. develop a Cu@Co‐MOF‐74 (Cu‐incorporated Co‐MOF‐74) catalyst that exhibits high activity and selectivity in nitrate reduction (Figure 5c). At −0.88 V vs. RHE, it delivers an ammonia production rate of 307.98 mmol/h/g_cat_ with a FE of 84.01% (Figure 5d). The synergistic effect of the interaction between the copper and cobalt sites increases the electron transfer and boosts NH_3_ yield and selectivity. This catalyst has been shown to be capable of sustainable ammonia synthesis based on 15N isotope labeling of the product NH_3_, which demonstrates that the NH_3_ produced is a result of the reduction of NO_3_^−^ [84].

Yang et al. reported that MOF‐derived copper catalysts dispersed on ceria/carbon, zirconium/carbon, and carbon catalyst were prepared as sacrificial templates (Figure 5e) from copper‐incorporated cerium‐based MOF, zirconium‐based metal‐organic framework (MOF), and copper‐based MOF as precursors, respectively. Assess Cu electrocatalysts derived from MOFs supported on various substrates (CeO_2_/carbon, ZrO/carbon, and carbon) for nitrate reduction. The selectivity of NH_3_ production varies from 47.2% of the carbon support to 36.0% of ZrO_2_/carbon support, and with the highest NH_3_ selectivity of 73.4% at −1.29 V vs. SHE, CeO_2_/carbon support is the best among the three supports. The experimental results and DFT calculations suggest that the electronic structure of Cu sites is modified by the ceria support, thus increasing the selectivity for ammonia production. The results underscore the importance of selecting the appropriate support for Cu‐based electrocatalysts to ensure high NO_3_^−^RR [160].

Zhou et al. prepared Cu/Co‐doped carbon catalysts from MOFs for nitrate conversion. The optimized Co_1_Cu_2_/NC/Ni cathode has a high removal rate of NO_3_^−^ (94.3%) and high FE of NH_4_^+^ (91.0%) after 90 min (Figure 5f). Mechanistic studies reveal that the NO_3_^−^ molecules are more readily adsorbed on the Cu sites, and reduction of NO_3_^−^ primarily occurs on the Cu sites, whereas the Co sites are involved in splitting H_2_O to provide protons for the formation of NH_4_^+^. Direct electron transfer and surface hydrogen atom‐mediated pathways to NO_3_RR are found to be equal by using electron paramagnetic resonance (EPR) and quenching experiments. The results in this work show the synergistic effect of the Cu–Co mechanism in the catalyst and provide insights for the development of efficient multicomponent NO_3_^−^ reduction systems [136].

Wang et al. report an Fe@MOF‐808 electrocatalyst functionalized with phosphate (Fe@MOF‐808‐P) for efficient nitrate‐to‐ammonia conversion. At −0.9 V vs. RHE in 0.5 M K_2_SO_4_, the catalyst achieves a 90.8 ± 0.5% FE for NH_3_, with an NH_3_ production rate of 420.4 ± 18.1 μmol h^−1^ cm^−2^ (specific activity: 1035.9 ± 20.1 mg_NH3_ h^−1^ mg_Fe_) (Figure 5g). Comparative tests show that phosphate doping accelerates the hydrogenation of NO_3_^−^, confirming Fe centers as the primary active sites. The catalyst also demonstrates excellent stability over repeated electrolysis cycles. These findings underscore phosphate functionalization as a means to enhance nitrate hydrogenation kinetics and highlight Fe@MOF‐808‐P as a promising material for sustainable ammonia synthesis [125].

Kumar et al. reported a CuFe bimetallic MOF (with Cu:Fe = 1:2) as an efficient dual electrocatalyst for nitrate reduction (NO_3_^−^RR) and hydrogen evolution (Figure 6a). The optimized CuF(1:2) MOF achieves an ammonia yield of 5.08 mmol h^−1^ cm^−2^ and FE of 87% in NO_3_^−^RR (Figure 6b), alongside efficient hydrogen evolution (overpotential 175 mV, Tafel slope 295 mV/dec). Chronoamperometry confirms stable catalytic performance over 24 h. These results highlight the CuFe(1:2) MOF as a promising next‐generation catalyst for ammonia synthesis and HER applications [115].

**FIGURE 6:**
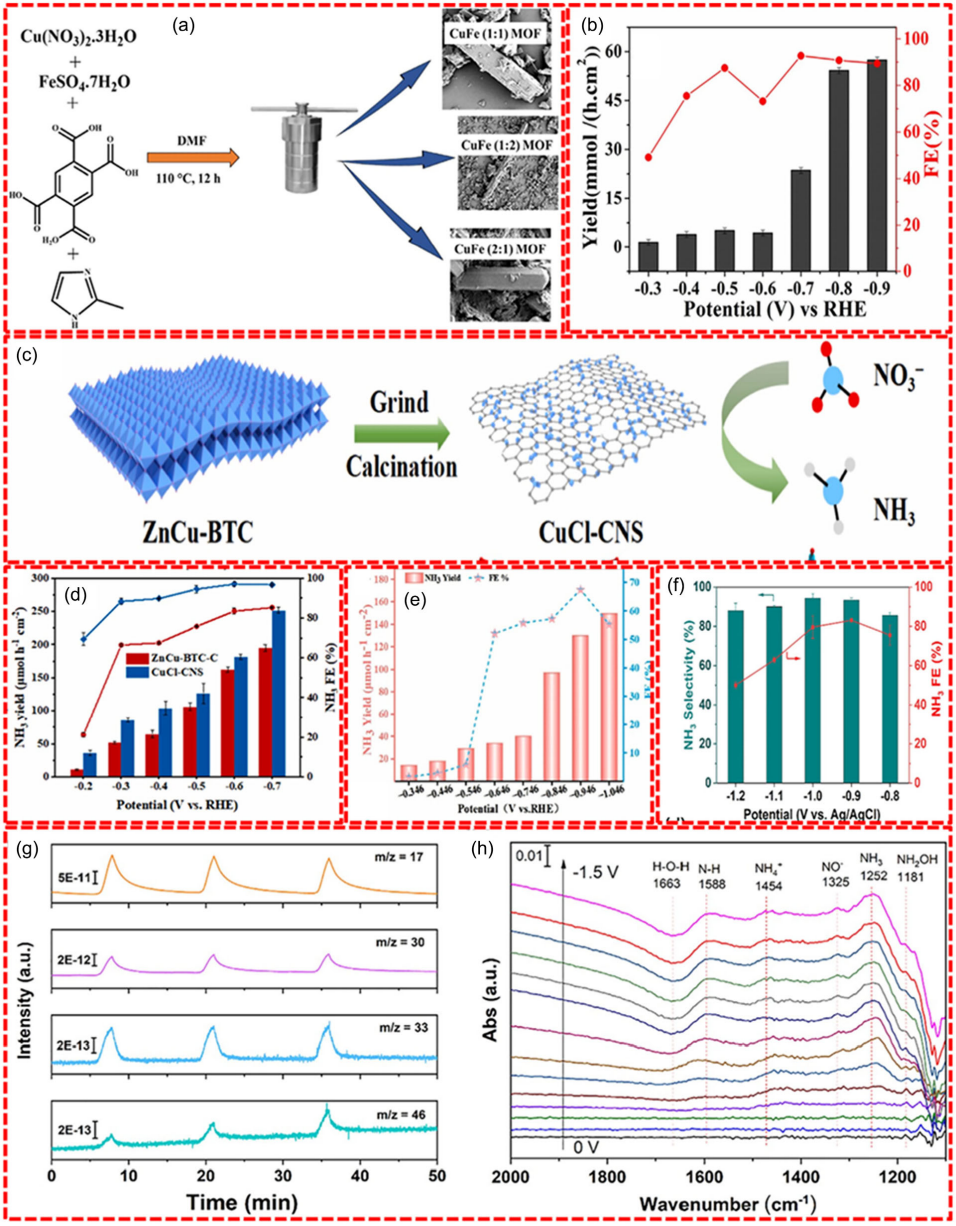
(a) preparation scheme of CuFe(x:y)MOF materials, (b) variation of NH_3_ yield and FE with applied potential for CuFe(1:2)MOF. Reproduced with permission from [115]. Copyright 2026, Elsevier. (c) Fabrication route of CuCl‐CNS designed for NO_3_^−^RR, (d) comparison of NH_3_ production rates and FEs for ZnCu‐BTC‐C and CuCl‐CNS. Reproduced with permission from [165]. Copyright 2024, Elsevier. (e) FEs and NH_3_ yields (M), including FE values for NO_3_^−^ → NO_2_^−^ and NO_3_^−^ →NH_3_ pathways Reproduced with permission from [166]. Copyright 2026, Elsevier. (f) NH_3_ selectivity and corresponding FE achieved over the CuNi/NC‐51 catalyst, (g) differential electrochemical mass spectrometry analysis of NO_3_^−^RR on CuNi/NC‐51, and (h) in situ SEIRAS spectra acquired during NO_3_^−^RR at various applied potentials Reproduced with permission from [135]. Copyright 2022, Elsevier.

Lin et al. synthesize porous carbon nanosheets (CNS) with CuCl nanoparticles by pyrolyzing the ZnCu‐BTC MOF in the presence of FeCl_3_ (Figure 6c). The electrocatalyst formed by the two components (CuCl‐CNS) shows high nitrate reduction activity, with NH_3_ FE of 96.9% and NH_3_ production rate of 292.5 μmol h^−^^1^ cm^−2^ at −0.60 V RHE (Figure 6d). This catalyst can be used in a zinc‐nitrate battery with a peak power density of 4.73 mW cm^−2^ and good long‐term cycling stability. The results of this study indicate that the Cu‐MOF‐derived nanocomposite material is an effective catalyst for nitrate reduction [165].

Wang et al. have reported the two‐dimensional Cu‐BDC (1,4‐benzenedicaboxylate) MOF as an electrocatalyst for NOx^−^ (NO_2_^−^/NO_3_^−^) reduction. In NO_2_^−^ reduction at −0.746 V vs. RHE, it yields NH_3_ at 71.20 μmol h^−1^ cm^−2^ (FE 80.9%), and in NO_3_^−^ reduction at −0.946 V, it yields 130.16 μmol h^−1^ cm^−2^ (FE 67.42%) (Figure 6e). The use of isotopic labeling, 15 NO_3_^−^, shows that the NH_4_^+^ produced is derived from nitrate. The NH_3_ yield and efficiency are well‐maintained during three cycles of NOx^−^RR, demonstrating the excellent stability of the catalyst under reaction conditions. The results demonstrate that the 2D Cu‐MOF is a promising material to study efficient nitrate/nitrite electroreduction [166].

To tackle the issue of the low activity of FeS_2_, Moa et al. have developed a MOF‐derived FeCoS_2_/Fe_0.95_S_1.05_ bimetallic sulfide catalyst for electrochemical nitrate reduction. The catalyst generates a high ammonia yield rate of 2.705 mg h^−1^ mg^−1^_cat_ with a high FE of 94.79% and high selectivity of ammonium (96.88%) with stable performance over 12 cycles. A theoretical and experimental study shows that Fe incorporation affects the electronic structure, favoring electron transfer from Fe to the adsorbed NO_3_^−^ . This makes it easier for NO_3_^−^ to be activated on Fe–Co sites and improves its conversion to NH_3_, resulting in a high catalytic ability. The study offers a guideline for the design of efficient nitrate reduction electrocatalysts of bimetallic sulfides [116].

Liu et al. synthesized CuNi alloy nanoparticles encapsulated in N‐doped carbon (CuNi/NC) by pyrolysis of the bimetallic MOFs. The CuNi/NC catalysts show significantly higher selectivity (94.4%) and FE (79.6%) for the conversion of nitrate to ammonia than the monometallic Cu/NC (selectivity: 60.8%) and Fe: 60.6%) catalysts (Figure 6f). Importantly, Ni incorporation inhibits the formation of the byproducts of nitrite. The key reaction intermediate, Ni, is detected by ifferential electrochemical mass spectrometry and SEIRS, and DFT calculations show that Ni changes the electronic structure of the catalyst (Figure 6g,h). The synergistic Cu–Ni sites facilitate the efficient hydrogenation of NO_2_ to HNO_2_ and suppress the HER, thus improving the selectivity of NH_3_ production. This work is also a novel synthetic approach and mechanistic understanding of the bimetallic nitrate reduction catalysts [135].

## 5 Conclusion

Electrocatalytic nitrate reduction to ammonia has attracted growing interest as a sustainable approach to mitigation of nitrate pollutants to valuable N chemicals. Compared with conventional nitrate removal processes, NO_3_^−^RR provides an opportunity to combine environmental remediation with nitrogen resource recovery. However, the reaction is still kinetically complex, due to the presence of several PCET steps, of intermediates containing N, and of pathways that result in unwanted products like nitrite (NO_2_^−^), nitrogen (N_2_), and hydrogen (H_2_) via the competing HER. Therefore, developing catalysts capable of controlling adsorption, activation, and subsequent hydrogenation processes is essential for achieving selective ammonia production. The modularity of MOFs has made them appealing catalyst platforms, allowing for systematic variation of metal sites, organic linkers, pore environment, and defect sites. Unlike traditional catalysts with relatively fixed structures, MOFs can be designed at the molecular level to create a microcatalytic environment. The electronic configuration of metal nodes, coordination environment, ligand functionality, and interface interaction are key factors that can be crucial in determining the catalytic performance of MOF‐based NO_3_^−^RR catalysts, which is discussed in this review. The structural factors control the adsorption of nitrate, electron transfer, proton transfer, and stabilization of intermediates such as *NO_2_ and *NO and can ultimately dictate ammonia selectivity. Recent studies have shown that rational modification strategies such as bimetallic incorporation, development of conductive frameworks, defect engineering, and carbon architecture derived from MOFs can overcome some of the limitations of pristine MOFs. More specifically, cooperative interactions in multinuclear active sites help to activate nitrate and intermediate hydrogenation, and MOF‐derived materials provide better electrical conductivity and robustness. Likewise, functionalized ligands and restricted pore environments can affect the local proton availability and intermediate binding, which can further regulate reaction pathways. However, there are some key fundamental and practical issues that have yet to be resolved. The internal electrical conducting properties of most MOFs are relatively low, which hinders rapid charge transport in electrochemical applications, and the stability of the MOF structure is also a concern during their long‐term electrochemical operation under aqueous conditions. MOF‐derived catalysts and hybrid systems have alleviated these drawbacks, but the change in the framework during operation can make it challenging to identify the real catalytic active phase. Moreover, reported performances are often measured under optimized laboratory conditions, and different electrolyte compositions, nitrate concentrations, applied potential, and ammonia quantification methods make it difficult to directly compare the performances of different catalyst systems.

## 6 Future Prospectus

While MOF‐based electrocatalysts have shown promising catalytic activity in the conversion of nitrate to ammonia, future research should be extended to improve the catalytic activity, which is not limited to the ammonia yield and FE. It is essential to have a more complete understanding of the evolution of the active sites during electrochemical operation as many MOF structures may reconstruct, partially degrade, or transform to derivative phases under reducing potentials. The elucidation of the true catalytic species and the understanding of their dynamic structural changes in relation to reaction pathways will be crucial for achieving meaningful structure–activity relationships. The rational design of the conductive MOF architecture is one key aspect. Many conventional MOFs have poor electrical conductivity that hinders electron transfer between the electrode and catalytic center, decreasing active site utilization. Future research should be directed toward the synthesis of intrinsically conductive MOFs, 2D frameworks, π‐conjugated ligands, and composite MOF‐based materials with hybridization of structural tunability and enhanced charge mobility. However, an increase in conductivity needs to be accompanied by the retention of the available active sites and framework stability. An additional important opportunity is the accurate control of active site environments. Future research for the development of catalysts should focus on controlling the coordination structure, electronic state, and spatial arrangement of the active centers, rather than on increasing the number of catalytic centers. Bimetallic and multinuclear architectures are among the most interesting as the cooperative effects among metals can, in principle, support independently both the activation of nitrate and the dissociation of water as well as intermediate hydrogenation. Ligand functionalization and defect engineering, on the other hand, could result in other methods of tuning adsorption energies and reaction selectivity. In future studies, more importance should be given to mechanistic studies. While the DFT‐based calculations proved to be useful to gain insights into adsorption energies and reaction barriers, operando experimental methods such as Raman spectroscopy, infrared spectroscopy, X‐ray absorption spectroscopy, and isotope‐labeling studies should be combined with the computations to gain deeper understanding. These can be useful for identifying transient intermediates and catalyst evolution during actual electrochemical processes, providing more accurate scientific guidelines towards catalyst design. Application‐wise, future studies on MOF‐based catalysts under real‐life conditions are required. The studies that are reported are performed with simplified nitrate‐containing electrolytes, while real wastewater matrices contain competing ions, organic contaminants, and changing nitrate concentrations. Hence, practical feasibility must be evaluated under realistic wastewater conditions, under continuous flow, and under high current‐density conditions. Further, ammonia quantification, nitrogen balance calculation, FE calculation, and stability testing should be standardized to enable the reliable comparison of different catalyst systems.

## Author Contributions

**Abdullah Shah:** investigation, formal analysis, and writing – original draft. **Muhammad Ilyas:** data curation, and conceptualization. **Natasha Shehzad:** formal analysis, and writing – original draft. **Shah Faisal:** formal analysis, data curation. **Salman Khan**: visualization, provided overall supervision. **Nolubabalo Matinise:** writing – review and editing, supervision, and project administration.

## Funding

The authors have nothing to report.

## Conflicts of Interest

The authors declare no conflicts of interest.

## Data Availability

No new data were generated in this review.
